# *De novo* PAM generation to reach initially inaccessible target sites for base editing

**DOI:** 10.1242/dev.201115

**Published:** 2023-01-23

**Authors:** Kaisa Pakari, Joachim Wittbrodt, Thomas Thumberger

**Affiliations:** ^1^ Centre for Organismal Studies Heidelberg (COS), Heidelberg University, Im Neuenheimer Feld 230, 69120 Heidelberg, Germany; ^2^ Heidelberg Biosciences International Graduate School (HBIGS), Heidelberg University, Im Neuenheimer Feld 501, 69120 Heidelberg, Germany

**Keywords:** Base editing, *De novo* PAM, Targeted mutagenesis, CRISPR, Medaka

## Abstract

Base editing by CRISPR crucially depends on the presence of a protospacer adjacent motif (PAM) at the correct distance from the editing site. Here, we present and validate an efficient one-shot approach termed ‘inception’ that expands the editing range. This is achieved by sequential, combinatorial base editing: *de novo* generated synonymous, non-synonymous or intronic PAM sites facilitate subsequent base editing at nucleotide positions that were initially inaccessible, further opening the targeting range of highly precise editing approaches. We demonstrate the applicability of the inception concept in medaka (*Oryzias latipes*) in three settings: loss of function, by introducing a pre-termination STOP codon in the open reading frame of *oca2*; locally confined multi-codon changes to generate allelic variants with different phenotypic severity in *kcnh6a*; and the removal of a splice acceptor site by targeting intronic sequences of *rx3*. Using sequentially acting base editors in the described combinatorial approach expands the number of accessible target sites by 65% on average. This allows the use of well-established tools with NGG PAM recognition for the establishment of thus far unreachable disease models, for hypomorphic allele studies and for efficient targeted mechanistic investigations in a precise and predictable manner.

## Teaser

**Summary:** A combinatorial and sequential one-shot approach termed ‘inception’ is a twist on base editing that allows initially inaccessible nucleotides to be reached.

## INTRODUCTION

The major drawback of conventional CRISPR/Cas9 targeted mutagenesis approaches is the unpredictable outcome caused by arbitrary non-homologous end-joining events that seal the introduced double-strand break (DSB). In contrast, base editors circumvent this ambiguity, as no DSBs are introduced and nucleotides in a defined base editing window are edited in a precise and predictable manner (Komor et al., 2016). In basic research, the nearly homozygous editing that is already present in the injected generation (F0) allows modeling of human diseases, hypomorphic allele studies and efficient mechanistic studies by altering codons for specific and functionally relevant amino acids (Cornean et al., 2022). Furthermore, the highly precise base editing becomes progressively more important for therapeutic applications, as apparent by the first clinical trials launched (Eisenstein, 2022).

The general applicability of base editing is constrained by the intrinsic geometry of the target site: a protospacer adjacent motif (PAM) sequence has to be located in the correct distance, i.e. 13-17 nucleotides downstream of the desired nucleotide target for proper base editing. One attempt to overcome this limitation currently being considered is the use of so-called (near) PAM-less (Walton et al., 2020) or PAM-free (Tan et al., 2022) base editors. However, their extended targeting range comes at the cost of reduced specificity and consequently enhanced off-target effects (Walton et al., 2020).

Here, we present an alternative, taking advantage of the well-established adenine and cytosine base editors [ABE8e (Richter et al., 2020), ancBE4max (Koblan et al., 2018) and evoBE4max (Thuronyi et al., 2019)] with NGG-PAM recognition to reach initially inaccessible sites for base editing. This is achieved by an initial *de novo* PAM generation (step 1) and the subsequent (step 2) base editing at the new site that we term ‘inception’.

## RESULTS AND DISCUSSION

**Fig. 1.**
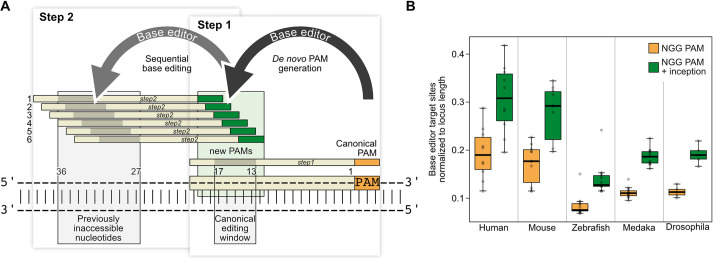
**Inception increases the base editing scope through *de novo* PAM generation.** (A) Schematic representation of the one-shot inception approach (simultaneous application of two base editors and two guide RNAs). If the adenosine(s) of NGA, NAA and NAG motifs are contained within the canonical base editing window, an A-to-G edit event leads to the generation of (up to six) new PAM(s) (green, step 1), rendering a new guide RNA target site available for a second base editing event (step 2) 27-36 nucleotides upstream of the canonical PAM (orange). As the second base editing relies on the first, the inception approach depicts a locally confined sequential editing event. (B) Abundance analysis of canonical base editor target sites with NGG PAM (orange) and target site increase upon inception approach (green) normalized to gene locus length. Base editor target sites contain A or C nucleotide(s) in the respective editing window. Comparison of the top ten studied human genes (*AKT1*, *APOE*, *EGFR*, *ESR1*, *IL6*, *MTHFR*, *TGFBI*, *TNF*, *TP53* and *VEGFA*) and their orthologs in commonly used model organisms (Table S1). Across all loci and organisms, inception increases accessible target sites by 64.8±6.0%. Boxplot shows median with boundaries representing the 25th and 75th percentiles. Whiskers extend to a maximum of 1.5 times the interquartile range. PAM, protospacer adjacent motif.

Based on a canonical guide RNA target site (*step1* guide RNA), a *de novo* PAM can be introduced by A-to-G base editing if the adenosine(s) of AA, GA or AG dinucleotides are contained within the canonical base editing window. Conditioned by this editing, a novel guide RNA target site becomes available for a second guide RNA (*step2* guide RNA)/base editor complex, subsequently introducing the intended mutation 27-36 nucleotides upstream of the original canonical PAM (Fig. 1A). The sequential nature of editing by the inception approach allows simultaneous application of all players: the A-to-G base editor combined with the canonical guide RNA to introduce the *de novo* PAM site (*step1*) as well as the second guide RNA (*step2*), and a possible additional base editor that binds the newly generated target site for the ultimate introduction of the intended edit(s). In other words, for a desired edit at a given position, a canonical NGG PAM site can be located anywhere within a distance of 27-36 nucleotides downstream. This flexibility relaxes the intrinsic constraints of a single target site while maintaining high targeting specificity. When comparing the top ten most studied human genes and their orthologs in commonly used model organisms, inception increases the number of editing sites by 65% on average (Fig. 1B, Table S1).

To address the applicability of the inception concept, we applied this sequential targeting approach in three different settings. Two settings were loss-of-function regimes: introduction of non-synonymous codon changes [including a pre-termination STOP codon (PTC) in an open reading frame]; or the removal of a splice acceptor site by targeting intronic sequences. Both these are conditions under which further codon changes are at least negligible. In the third setting, we used inception to introduce locally confined predictable multi-codon changes to generate allelic variants with different phenotypic severity.

**Fig. 2.**
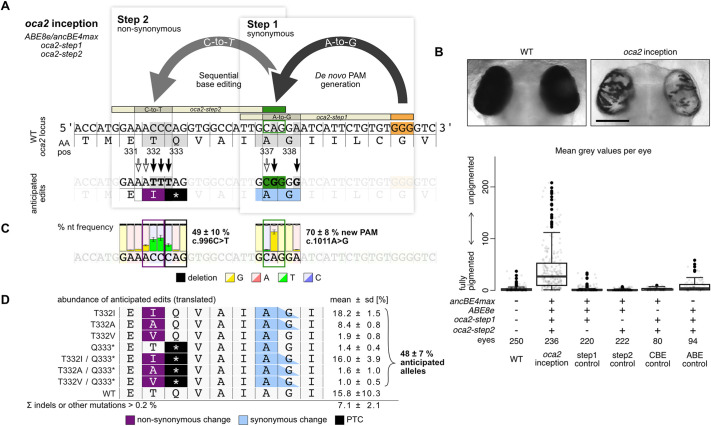
**Inception efficiently introduces loss-of-pigmentation mutations in *oca2*.** (A) Loss-of-function mutations via inception at the *oca2* locus by synonymous *de novo* PAM generation in step 1 (ABE8e, CAG>CGG=p.A337A) and subsequent non-synonymous editing (ancBE4max, CAG>TAG=p.Q333* and ACC>ATT=p.T332I) to introduce non-synonymous codon changes, including a pre-termination STOP codon (PTC) at p.T332 and p.Q333 in step 2. Besides anticipated edits (black arrows), potential further edits (white arrows) may occur due to combined injection of A-to-G and C-to-T base editors (compare with Fig. S1). (B) At 4.5 days post-fertilization, full pigmentation of wild-type *Oryzias latipes* eyes (left) is lost in *oca2* inception editants (right), as quantified by analysis of mean gray values per eye. The *oca2*-inception injection mix contained ABE8e and ancBE4max mRNA, and *oca2-step1* and *oca2-step2* guide RNAs. Each control mix lacked one component and injections did not show spurious activity. In the case of the ABE control, mild effects through editing T332A matched our previous report (Cornean et al., 2022), compare with Fig. S2. Boxplot shows median with boundaries representing the 25th and 75th percentiles. Whiskers extend to a maximum of 1.5 times the interquartile range. Scale bar: 200 μm. (C) Illumina amplicon sequencing reveals a high abundance of anticipated edits. The nucleotide abundance is displayed as the mean±s.d. of three replicates (pools of eight phenotypic embryos each; 246,045 reads total; Fig. S2). (D) Frequency analysis of resulting alleles (translated) highlights anticipated edits to reach 48.4±6.7% abundance. Analysis is based on CRISPResso2 Alleles frequency table with cut-off at >0.2% Illumina sequence read abundance per replicate (Fig. S3). AA pos, amino acid position; nt, nucleotide; PAM, protospacer adjacent motif; WT, wild type. Orange indicates canonical PAM; green indicates *de novo* PAM.

To validate the efficiency of a knockout via inception, we targeted the well-described *oculocutaneous albinism 2* (*oca2*) gene responsible for the pigmentation of the retinal pigmented epithelium (RPE) in the Japanese rice fish medaka (*Oryzias latipes*) (Cornean et al., 2022; Lischik et al., 2019). The loss of pigmentation depends on bi-allelic editing of the *oca2* gene, which we use as a proxy to determine knockout efficiency via an established analysis pipeline (Thumberger et al., 2022). Using base editors, we recently demonstrated that, in *oca2*, non-synonymous changes of threonine 332 (T332), as well as the introduction of a PTC (glutamine>PTC, Q333*) resulted in substantial loss of pigmentation (Cornean et al., 2022). As proof of concept, we targeted these codons with the inception approach, in which the CAG motif downstream of Q333 can serve as a *de novo* PAM site upon A-to-G editing (adenine c.1011), leading to a synonymous edit (A337A; Fig. 2A). For the *oca2* inception approach, two guide RNAs were selected: the canonical *oca2-step1* guide RNA to generate the novel PAM site in combination with the A-to-G base editor (ABE8e); and the *oca2-step2* guide RNA in combination with the C-to-T base editor (ancBE4max) to introduce the anticipated T332I and Q333* edits (Fig. 2A; black arrows). As the two base editing windows contain target nucleotides for both base editors, further edits may occur (Fig. 2A, white arrows; Fig. S1).

Upon injection of the *oca2*-inception mix (*ABE8e*, *ancBE4max*, *oca2-step1* guide RNA and *oca2-step2* guide RNA) into one-cell stage medaka embryos, efficient loss of RPE pigmentation was detected at 4.5 days post-fertilization (Fig. 2B; Table S2). PCR amplification and Illumina sequencing of the targeted *oca2* locus (three pools of eight editants; 246,045 reads total) revealed efficient *de novo* PAM generation (c.1011A>G) in 70.3±8.3% alleles and a high subsequent editing efficiency in the second base editing window of up to 48.5±10.1% alleles at the codon of T332 (c.996C>T) and 23.0±5.8% alleles at the neighboring codon of Q333 (c.997C>T; Fig. 2C, Fig. S2). Comparing the C-to-T conversion rate in the codon of Q333 of our inception approach with our previously published direct base editing (Cornean et al., 2022), total nucleotide edits amounted to 23.0% versus 65.3%, respectively. Considering that our *de novo* PAM generation is 70.3%, the effective inception editing rate is still 33%. It is noteworthy that low level editing outside the general base editing window can occur (Cornean et al., 2022).

Frequency analysis of the resulting alleles predominantly revealed the intended synonymous changes at the *de novo* PAM site that facilitated subsequent non-synonymous editing at the codons T332 and Q333. Nearly half of all alleles analyzed (48.4±6.7%) contained the anticipated loss-of-function codon changes, i.e. 28.6±1.7% harbored a single T332 codon change and 19.9±5.0% contained the Q333* mutation (Fig. 2D, Fig. S3). All these alleles contained a *de novo* PAM that resulted in A337A synonymous codon change. Wild-type alleles were detected at a rate of 15.8±10.3%. Unwanted on-target substitutions were rarely generated and indels were also found at low rates (7.1±2.1%; Fig. 2D; Fig. S3).

Strikingly, although the inception mix contained adenine and cytosine base editors that could edit at both sites (Fig. 2A, white arrows; Fig. S1), the highest activity was detected at the respective intended target site (Fig. 2A, black arrows; Fig. S2). This is best explained by the dinucleotide context, i.e. the influence of the preceding nucleotide on the editing efficiency. In the case of the ABE8e, a preceding adenine, as present in the E331/T332 codons, can drastically reduce the editing efficiency (Cornean et al., 2022). The same is true for the canonical base editing window in which the cytosine of codon A337 (GCA) is preceded by a guanine that reduces the efficiency of the ancBE4max (Cornean et al., 2022). Thus, both base editors performed most efficiently at the intended target sites.

We could not detect spurious activity of base editors and guide RNAs, as addressed in control injections with both editors and either one of the two guide RNAs (Fig. 2B, Table S2; step1 control, step2 control). Illumina sequencing of the *oca2-step1* control injection (three pools of one to eight editants, 82,182 reads total) revealed 80.6±7.3% alleles with *de novo* PAM generation (c.1011A>G, Fig. S2) across all three replicates; the pigmentation was no different from wild-type. The *oca2-step2* control injection (three pools of eight editants; 124,571 reads total) underscored that, in the absence of a NGG PAM, editing was highly inefficient (maximum of 1.2±0.8% of alleles with c.995C>T) (Fig. S2).

As further controls, both guide RNAs were co-injected with either base editor. In the case of the CBE control, this yielded neither an apparent loss of pigmentation nor a change in the nucleotide sequence (pool of eight editants) (Fig. S2). Expectedly, the ABE control showed a mild effect, following the introduction of the *de novo* PAM and subsequent editing of T332A (pool of five editants, Fig. S2), as reported before by conventional A-to-G base editing (Cornean et al., 2022). In summary, we demonstrate that the combinatorial use of sequentially acting base editors in the inception approach is predictable and highly efficient to render *oca2* non-functional.

**Fig. 3.**
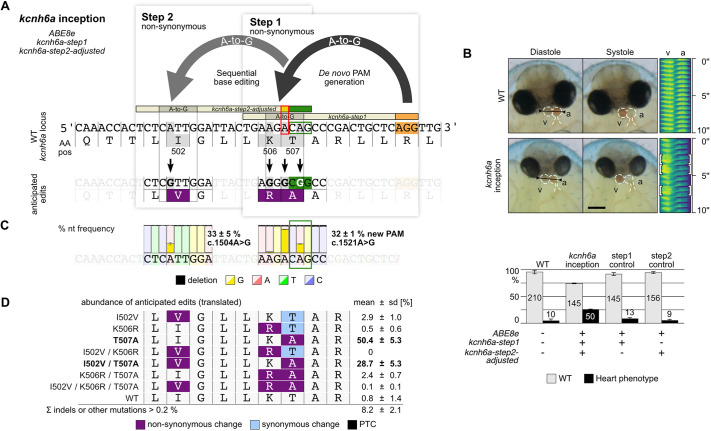
**Inception efficiently introduces locally confined multi-codon changes in *kcnh6a*.** (A) Local multi-codon editing via inception (both non-synonymous; anticipated edits are indicated by black arrows) at the *kcnh6a* locus to create hypomorphic alleles with ABE8e and two guide RNAs [*kcnh6a-step1* and sequence adjusted (red outlined box) *kcnh6a-step2-adjusted*]. (B) At 4 days post-fertilization, the two-chambered embryonic wild-type *Oryzias latipes* heart displays strong diastole/systole periodicity [regularly patterned kymograph; the line used spans the ventricle (v) and atrium (a), indicated in left panel]. *kcnh6a-*inception editants displayed heart phenotypes (26.9%), including heart morphology, reduced ventricular contractility and the exemplary displayed 2:1 atrioventricular block [white brackets in kymograph (line indicated in left panel; Movie 1)]. Scale bar: 200 μm. Control injections were wild type-like (barplot; data are mean±s.d. of three replicates, *n*=number of embryos). (C) Illumina amplicon sequencing reveals high abundance of anticipated edits. The nucleotide abundance is displayed as the mean±s.d. of three replicates (pools of five to ten embryos each; 164,108 reads total; Fig. S6). (D) Frequency analysis of resulting alleles (translated) comparing the anticipated single, double and triple codon changes. Analysis based on CRISPResso2 Alleles frequency table with cut-off at >0.2% Illumina sequence read abundance per replicate (Fig. S7). AA pos, amino acid position; nt, nucleotide; PAM, protospacer adjacent motif; WT, wild type. Red box indicates A>G adjustment in *kcnh6a-step2-adjusted* guide RNA; orange indicates canonical PAM; green indicates *de novo* PAM.

Loss-of-function studies provide valuable insight into the organismal response to the lost gene function but are often accompanied by early lethality and are not always conclusive. Studying hypomorphs might overcome the lethality problem of null mutants, especially in developmental or cellular key genes (Peterson and Murray, 2022). We targeted the *kcnh6a* gene [*potassium voltage-gated channel, subfamily H (eag-related), member 6a*], a key gene controlling heart contraction, in its highly conserved and mutation-sensitive membrane-spanning S4 domain (Cornean et al., 2022; Hoshijima et al., 2019). Multiple non-synonymous codon substitutions allow the correlation of structural changes with the severity of the resulting phenotypes. To accumulate locally confined multi-codon edits, we designed a pair of guide RNAs for the A-to-G base editor to introduce a new PAM that should cause K506R/T507A (*kcnh6a-step1*) and the sequential facilitation of I502V (*kcnh6a-step2*) substitutions by inception (Fig. 3A, Fig. S4). For efficient binding of the *kcnh6a-step2* guide RNA, it is important to consider changes introduced in the canonical base editing window in step 1. This requires sequence adjustment of the *kcnh6a-step2* guide RNA (*kcnh6a-step2-adjusted*) to bind and facilitate subsequent editing. Without adjustment, injection of the wild-type *kcnh6a* inception mix (*ABE8e*, *kcnh6a-step1* and *kcnh6a-step2-wt*) did not lead to editing of I502V at step 2 (Fig. S5). This is explained by the prevention of sequential editing of I502V by the c.1519A>G edit introduced in step 1 (Fig. S5). This nucleotide demarcates the first position of the *kcnh6a-step2* guide RNA target sequence (c.1519A red box, Fig. S5) and Cas9 enzymes do not tolerate PAM-proximal mis-matches (Hsu et al., 2013). Consequently, injection of the *kcnh6a*-inception mix containing the *kcnh6a-step2-adjusted* guide RNA resulted in 25.5±1.3% heart phenotypes (Table S2), comprising 2:1 atrioventricular block and reduced ventricular contractility, similar to earlier reports (Cornean et al., 2022) (Fig. 3B, Movie 1). Illumina sequencing of phenotypic editants (three pools of five to ten editants; 164,108 reads total) confirmed efficient *de novo* PAM generation (32.3±1.3% alleles, c.1521A>G) and highly efficient subsequent editing in the second base editing window (c.1504A>G with 33.3±4.9% alleles; Fig. 3C, Fig. S6). Frequency analysis of the resulting alleles predominantly revealed the intended non-synonymous T507A codon change, whereas the anticipated K506R was under-represented (Fig. 3D, Fig. S7). The inefficient K506R codon change (AAC>AGC) can again be explained by the AA dinucleotide context: the preceding adenine of the anticipated edited nucleotide (c.1504A>G) has a strong negative impact on the ABE8e employed. Injection of the control mixes caused phenotypes in only rare cases (Fig. 3B). Although the *de novo* PAM site mutation was efficiently introduced (c.1521A>G, 37.3±8.1% alleles, three pools of five editants; 103,384 reads total; Fig. S6) in the *kcnh6a-step1* control, phenotypes were low (8.3±3.1%; Table S2). The *kcnh6a-step2-adjusted* did not result in scorable editing events (0.1±0.1%) in the absence of the canonical editing event (Fig. S6; three pools of five editants; 91,445 reads total). Taken together, this highlights the potential of the inception approach to introduce a range of precisely targeted alterations in putatively critical functional domains for systematic structure-function analyses.

**Fig. 4.**
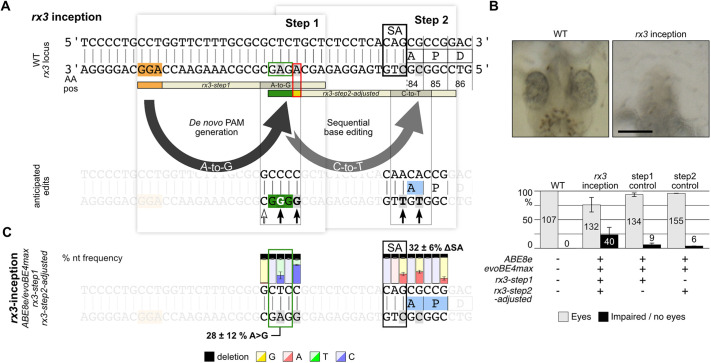
**Efficient splice site acceptor removal via inception in *rx3*.** (A) A splice acceptor site mutation (CAG>CAA, black outlined box) in *rx3* via intronic *de novo* PAM generation (*rx3-step1* guide RNA, ABE8e; GAG>GGG) and subsequent C-to-T splice acceptor site editing [sequence adjusted *rx3-step2-adjusted* guide RNA (red outlined box), evoBE4max; CAG>CAA]. (B) At stage 26, *Oryzias latipes* eye development is apparent in wild-type embryos (left), but drastically affected in *rx3-*inception editants (right, 23.9±13.4%) and rarely found in control injections (barplot; data are mean±s.d. of three replicates; *n*=number of embryos). Scale bar: 200 μm. (C) Illumina amplicon sequencing reveals the high rates of the anticipated splice acceptor mutation (32.0±6.1%). The nucleotide abundance is displayed as the mean±sd of three replicates (pools of five to nine embryos each; 136,642 reads total; Fig. S6). AA pos, amino acid position; nt, nucleotide; PAM, protospacer adjacent motif; SA, splice acceptor; WT, wild type. Red box indicates sequence adjustment in *rx3-step2*-adjusted guide RNA; orange indicates canonical PAM; green indicates *de novo* PAM.

In our third approach, we generated splice site mutations to interfere with gene function (García-Tuñón et al., 2019). We used inception to target the splice acceptor site of coding exon 2 of the *retinal homeobox transcription factor 3* gene (*rx3*) that is required for proper optic vesicle evagination. Rx3 mutants exhibit severe retinal phenotypes ranging from anophthalmia to microphthalmia (Loosli et al., 2001; Zilova et al., 2021). We designed a guide RNA by which the *de novo* PAM is introduced in the intronic sequence upstream of exon 2 (*rx3-step1* guide RNA) and a second guide RNA targeting the CAG splice acceptor site and first codons of exon 2 (Fig. 4A, Fig. S8). As the first nucleotide (adenine) of the *rx3-step2* guide RNA is contained within the canonical base editing window, adjustment of the *rx3-step2* guide RNA sequence (red box, Fig. 4A) is required, as detailed above for *kcnh6a*. In 23.9±13.4% of the injected *rx3-*inception editants, eyes were lost or dramatically underdeveloped (Fig. 4B; Table S2). Illumina sequencing (three pools of five to nine editants; 136,642 reads total) revealed efficient *de novo* PAM generation (27.6±12.5% alleles) and subsequent mutation of the CAG splice acceptor site to CAA (32.0±6.1% alleles, Fig. 4C, Fig. S9 and Fig. S10). In the *rx3-step1* control injections, editants showed low rates of impaired eye development (6.3±3.6% alleles) best explained by a prominent indel formation along the *rx3-step1* guide RNA target site reaching up to 36.7%, as revealed by Illumina sequencing (three pools of five editants; 97,976 reads total; Fig. S9). Injections of the *rx3-step2-adjusted* control resulted in a low rate of impaired eye development in 3.8±0.6% alleles, correlating with low rates of SA mutations (3.4±2.6%), as revealed by Illumina sequencing (three pools of five editants; 82,283 reads total; Fig. S9). Taken together, the introduction of an intronic *de novo* PAM site allows efficient sequential manipulation of a splice acceptor site via inception, resembling the phenotypes of established *rx3* mutants.

Overall, inception allows the prominent extension of the canonical editing range, as detailed in three different contexts. The efficient sequential editing in a one-shot approach not only allows unreachable sites to be edited but also facilitates the efficient mechanistic probing of putatively functional protein domains. The sequential combination of base editors relying on the presence of a NGG PAM site, and thus the extension of the editing range, does not come at the price of relaxed stringency at the level of the guide RNAs. Our detailed analysis by Illumina sequencing highlights the particular relevance of the consideration of the dinucleotide context when ‘designing’ the preferred edits. By introducing multiple codon changes (allelic variants) or loss-of-function mutations, the impact of putative bystander mutations was not prominently apparent, providing a wider range of flexibility in selecting pairs of guide RNAs. The rate-limiting step for inception always was the *de novo* PAM generation. Once established, the second edit occurred almost quantitatively. Any first editing event introducing a new PAM site presents a substrate for inception. This raises unlimited possibilities regarding combinations of different PAMs and base editors, which broadens the targeting range without compromising target specificity.

## MATERIALS AND METHODS

### Fish maintenance

Adult medaka fish (*Oryzias latipes,* Cab strain) were bred and maintained as closed stocks at 28°C on a 14h:10h light:dark cycle at Heidelberg University. Fish husbandry and experiments were performed in accordance with the local animal welfare standards (Tierschutzgesetz §11, Abs. 1, Nr. 1, husbandry permit number 35-9185.64/BH Wittbrodt).

### Base editor plasmids and mRNA synthesis

The following plasmids were used in this study: pCS2+_evoBE4max (Cornean et al., 2022), pCMV_AncBE4max (Addgene 112094) and pCMV_ABE8e (Addgene 138489) were gifts from David Liu.

ABE8e and ancBE4max plasmids were linearized with SapI (New England Biolabs) and the evoBE4max plasmid was digested with NotI-HF (New England Biolabs). The digests were purified using the QIAquick PCR Purification Kit (Qiagen). *In vitro* transcriptions of mRNAs were performed with the mMESSAGE mMACHINE SP6 or T7 Transcription Kit (Thermo Fisher Scientific) and purified with the RNeasy Mini Kit (Qiagen), according to manufacturers' protocols. The quality of the mRNA was assessed with a RNA test gel.

### sgRNAs and crRNAs

All guide RNAs (*oca2*, *rx3* and *kcnh6a*) were checked for off-targets using CCTop (Stemmer et al., 2015) and ACEofBASES (Cornean et al., 2022) with standard parameters. Guide RNAs used in this work are listed in Table S3. Cloning of single guide RNA (sgRNA) templates and transcription was performed as described previously (Stemmer et al., 2015). The plasmid DR274 was a gift from Keith Joung (Addgene 42250) (Hwang et al., 2013).

Target-specific crRNAs and tracrRNA were ordered from IDT (custom Alt-R crRNA). crRNA (100 µM) and tracrRNA (100 µM) were diluted in nuclease-free duplex buffer (IDT) to a final concentration of 40 µM and incubated at 95°C for 5 min.

### Microinjections

Microinjections were performed in wild-type Cab embryos at the one-cell stage. Fertilized embryos were injected with the injection mix listed in Table S2. After injections, embryos were kept in embryo-rearing medium (1× ERM: 17 mM NaCl, 40 mM KCl, 0.27 mM CaCl2•2H2O, 0.66 mM MgSO4•7H2O and 17 mM HEPES) and incubated at 26°C or 18°C for *rx3*-targeted editants. Embryos were screened for GFP expression 6 h or 1 day after injection on a Nikon SMZ18 stereomicroscope. Only GFP-positive and properly developed embryos were continued with (Table S2).

### Image acquisition and phenotyping

For analysis of *oca2* knockouts, the embryos were fixed 4.5 days post-fertilization (dpf) (Iwamatsu, 2004) in 4% paraformaldehyde in 1× PBS (137 mM NaCl, 2.7 mM KCl, 240 mg/l KH_2_PO_4_ and 1.44 g/l Na2HPO4). Images of the eyes of properly developed embryos were acquired with the ACQUIFER Imaging Machine (DITABIS) and the mean gray value per eye was quantified as previously described (Thumberger et al., 2022).

Embryos injected with guide RNAs targeting *rx3* and *kcnh6a* were imaged at 4 dpf or 9 dpf with a Nikon digital DS-Ri1 camera mounted onto a Nikon Microscope SMZ18 equipped with the Nikon Software NIS-Elements F version 4.0.

### Genotyping and targeted amplicon sequencing by Illumina

For genotyping via Illumina sequencing, embryos were ground and lysed in DNA extraction buffer [0.4 M Tris/HCl (pH 8.0), 0.15 M NaCl, 0.1% SDS, 5 mM EDTA (pH 8.0); 1 mg/ml proteinase K) at 60°C overnight. Samples were diluted 1:2 with nuclease-free water and proteinase K was heat inactivated at 95°C for 20 min.

For *oca2* inception (three replicates, eight phenotypic editants each), *oca2-step2* control (three replicates, eight randomly picked editants each) and *oca2-step1* control (three replicates, one to eight embryos each) were processed for genotyping. For *kcnh6a* inception (three replicates, five to ten phenotypic editants each), *kcnh6a-step1* control and *kcnh6a-step2-adjusted* control (three replicates, five randomly picked editants each) were processed. For *rx3* inception (three replicates, five to nine phenotypic editants each), *rx3-step1* control and *rx3-step2-adjusted* control (three replicates, five randomly picked embryos each) were processed.

The three targeted regions of *oca2*, *kcnh6a* and *rx3* were PCR amplified with Q5 polymerase (New England Biolabs) and locus-specific primers 5′ extended with partial Illumina adapter sequences (Table S4). PCR products were extracted with the Monarch DNA Gel Extraction Kit (New England Biolabs) after running on an agarose gel. Samples genotyped by Illumina based amplicon sequencing were prepared by pooling multiple amplicons into a single reaction. PCR products from each locus were pooled to equimolarity at 20 ng/µl and submitted to GeneWiz (Azenta Life Sciences) for sequencing (Amplicon-EZ: Illumina MiSeq, 2×250 bp sequencing, paired end). Sequencing data were analyzed using CRISPResso2 v.2.1.2 (Clement et al., 2019), CRISPRessoPooled tool in Amplicon Mode. Default parameters were used for analysis except for quantification_window_center (17), plot_window_size (25) and quantification_window_size (50). Plotting of nucleotide abundance was performed in R v3.6.3 in Rstudio. The average percentages of nucleotide abundance and indel frequency of three replicates across the amplicons were calculated from the CRISPResso2 tool Nucleotide frequency table output file by calculating nucleotide/reads aligned or Indels/reads aligned. Allele frequencies were aligned and translated in Geneious Prime (2019.2.3, BioMatters) based on CRISPResso2 Alleles frequency table output files with cut off at 0.2% of reads per replicate.

### Genotyping via Sanger sequencing

For genotyping, up to eight embryos were ground and lysed in DNA extraction buffer, as detailed above. For *oca2-ABE* control injection, a pool of five phenotypic embryos were genotyped. For the *oca2-CBE* control injection, a pool of eight randomly picked editants were genotyped. For the *kcnh6a-step2-wt* injection, a pool of eight embryos was picked for genotyping.

Samples were PCR amplified using Q5 High-Fidelity DNA Polymerase (New England Biolabs) and locus-specific primers (Table S5), 1 µl DNA sample and 30 PCR cycles. PCR products were gel purified after agarose gel electrophoresis with Monarch DNA Gel Extraction Kit (New England Biolabs) and submitted for Sanger sequencing to Eurofins Genomics. The results were analyzed with EditR (1.0.10) (Kluesner et al., 2018).

### Data visualization

Microscopy images were processed using Fiji (Schindelin et al., 2012). Data visualization and analysis were performed with ggplot2 (Hadley, 2016) in RStudio 2022.2.2.485 (Team, 2022) and Geneious Prime (2019.2.3, BioMatters). Figures were assembled in Affinity Designer (1.10.5, Serif).

## Supplementary Material

10.1242/develop.201115_sup1 Supplementary information

## References

[DEV201115C1] Clement, K., Rees, H., Canver, M. C., Gehrke, J. M., Farouni, R., Hsu, J. Y., Cole, M. A., Liu, D. R., Joung, J. K., Bauer, D. E. et al. (2019). CRISPResso2 provides accurate and rapid genome editing sequence analysis. *Nat. Biotechnol.* 37, 224-226. 10.1038/s41587-019-0032-3 30809026 PMC6533916

[DEV201115C2] Cornean, A., Gierten, J., Welz, B., Mateo, J. L., Thumberger, T. and Wittbrodt, J. (2022). Precise in vivo functional analysis of DNA variants with base editing using ACEofBASEs target prediction. *Elife* 11, e72124. 10.7554/eLife.72124 35373735 PMC9033269

[DEV201115C3] Eisenstein, M. (2022). Base editing marches on the clinic. *Nat. Biotechnol.* 40, 623-625. 10.1038/s41587-022-01326-x 35534557

[DEV201115C4] García-Tuñón, I., Alonso-Pérez, V., Vuelta, E., Pérez-Ramos, S., Herrero, M., Méndez, L., Hernández-Sánchez, J. M., Martín-Izquierdo, M., Sáldana, R., Sevilla, J. et al. (2019). Splice donor site sgRNAs enhance CRISPR/Cas9-mediated knockout efficiency. *PLoS One* 14, e0216674. 10.1371/journal.pone.0216674 31071190 PMC6508695

[DEV201115C5] Hadley, W. (2016). *Ggplot2*. New York, NY: Springer Science+Business Media, LLC.

[DEV201115C6] Hoshijima, K., Jurynec, M. J., Klatt Shaw, D., Jacobi, A. M., Behlke, M. A. and Grunwald, D. J. (2019). Highly efficient CRISPR-Cas9-based methods for generating deletion mutations and F0 embryos that lack gene function in zebrafish. *Dev. Cell* 51, 645-657.e644. 10.1016/j.devcel.2019.10.004 31708433 PMC6891219

[DEV201115C7] Hsu, P. D., Scott, D. A., Weinstein, J. A., Ran, F. A., Konermann, S., Agarwala, V., Li, Y., Fine, E. J., Wu, X., Shalem, O. et al. (2013). DNA targeting specificity of RNA-guided Cas9 nucleases. *Nat. Biotechnol.* 31, 827-832. 10.1038/nbt.2647 23873081 PMC3969858

[DEV201115C8] Hwang, W. Y., Fu, Y., Reyon, D., Maeder, M. L., Tsai, S. Q., Sander, J. D., Peterson, R. T., Yeh, J. R. and Joung, J. K. (2013). Efficient genome editing in zebrafish using a CRISPR-Cas system. *Nat. Biotechnol.* 31, 227-229. 10.1038/nbt.2501 23360964 PMC3686313

[DEV201115C9] Iwamatsu, T. (2004). Stages of normal development in the medaka Oryzias latipes. *Mech. Dev.* 121, 605-618. 10.1016/j.mod.2004.03.012 15210170

[DEV201115C10] Kluesner, M. G., Nedveck, D. A., Lahr, W. S., Garbe, J. R., Abrahante, J. E., Webber, B. R. and Moriarity, B. S. (2018). EditR: a method to quantify base editing from sanger sequencing. *CRISPR J* 1, 239-250. 10.1089/crispr.2018.0014 31021262 PMC6694769

[DEV201115C11] Koblan, L. W., Doman, J. L., Wilson, C., Levy, J. M., Tay, T., Newby, G. A., Maianti, J. P., Raguram, A. and Liu, D. R. (2018). Improving cytidine and adenine base editors by expression optimization and ancestral reconstruction. *Nat. Biotechnol.* 36, 843-846. 10.1038/nbt.4172 29813047 PMC6126947

[DEV201115C12] Komor, A. C., Kim, Y. B., Packer, M. S., Zuris, J. A. and Liu, D. R. (2016). Programmable editing of a target base in genomic DNA without double-stranded DNA cleavage. *Nature* 533, 420-424. 10.1038/nature17946 27096365 PMC4873371

[DEV201115C13] Lischik, C. Q., Adelmann, L. and Wittbrodt, J. (2019). Enhanced in vivo-imaging in medaka by optimized anaesthesia, fluorescent protein selection and removal of pigmentation. *PLoS One* 14, e0212956. 10.1371/journal.pone.0212956 30845151 PMC6405165

[DEV201115C14] Loosli, F., Winkler, S., Burgtorf, C., Wurmbach, E., Ansorge, W., Henrich, T., Grabher, C., Arendt, D., Carl, M., Krone, A. et al. (2001). Medaka eyeless is the key factor linking retinal determination and eye growth. *Development* 128, 4035-4044. 10.1242/dev.128.20.4035 11641226

[DEV201115C15] Peterson, K. A. and Murray, S. A. (2022). Progress towards completing the mutant mouse null resource. *Mamm. Genome* 33, 123-134. 10.1007/s00335-021-09905-0 34698892 PMC8913489

[DEV201115C16] Richter, M. F., Zhao, K. T., Eton, E., Lapinaite, A., Newby, G. A., Thuronyi, B. W., Wilson, C., Koblan, L. W., Zeng, J., Bauer, D. E. et al. (2020). Phage-assisted evolution of an adenine base editor with improved Cas domain compatibility and activity. *Nat. Biotechnol.* 38, 883-891. 10.1038/s41587-020-0453-z 32433547 PMC7357821

[DEV201115C17] Schindelin, J., Arganda-Carreras, I., Frise, E., Kaynig, V., Longair, M., Pietzsch, T., Preibisch, S., Rueden, C., Saalfeld, S., Schmid, B. et al. (2012). Fiji: an open-source platform for biological-image analysis. *Nat. Methods* 9, 676-682. 10.1038/nmeth.2019 22743772 PMC3855844

[DEV201115C18] Stemmer, M., Thumberger, T., Del Sol Keyer, M., Wittbrodt, J. and Mateo, J. L. (2015). CCTop: an intuitive, flexible and reliable CRISPR/Cas9 target prediction tool. *PLoS One* 10, e0124633. 10.1371/journal.pone.0124633 25909470 PMC4409221

[DEV201115C19] Tan, J., Zeng, D., Zhao, Y., Wang, Y., Liu, T., Li, S., Xue, Y., Luo, Y., Xie, X., Chen, L. et al. (2022). PhieABEs: a PAM-less/free high-efficiency adenine base editor toolbox with wide target scope in plants. *Plant Biotechnol. J.* 20, 934-943. 10.1111/pbi.13774 34984801 PMC9055815

[DEV201115C20] Team, R. (2022). RStudio: Integrated Development Environment for R.

[DEV201115C21] Thumberger, T., Tavhelidse-Suck, T., Gutierrez-Triana, J. A., Cornean, A., Medert, R., Welz, B., Freichel, M. and Wittbrodt, J. (2022). Boosting targeted genome editing using the hei-tag. *Elife* 11, e70558. 10.7554/eLife.70558 35333175 PMC9068219

[DEV201115C22] Thuronyi, B. W., Koblan, L. W., Levy, J. M., Yeh, W. H., Zheng, C., Newby, G. A., Wilson, C., Bhaumik, M., Shubina-Oleinik, O., Holt, J. R. et al. (2019). Continuous evolution of base editors with expanded target compatibility and improved activity. *Nat. Biotechnol.* 37, 1070-1079. 10.1038/s41587-019-0193-0 31332326 PMC6728210

[DEV201115C23] Walton, R. T., Christie, K. A., Whittaker, M. N. and Kleinstiver, B. P. (2020). Unconstrained genome targeting with near-PAMless engineered CRISPR-Cas9 variants. *Science* 368, 290-296. 10.1126/science.aba8853 32217751 PMC7297043

[DEV201115C24] Zilova, L., Weinhardt, V., Tavhelidse, T., Schlagheck, C., Thumberger, T. and Wittbrodt, J. (2021). Fish primary embryonic pluripotent cells assemble into retinal tissue mirroring in vivo early eye development. *Elife* 10, e66998. 10.7554/eLife.66998 34252023 PMC8275126

