# Supplementary material for: De novo PAM generation to reach initially inaccessible target sites for base editing
Source: Development. 2023 Jan 23;150(2):dev201115. doi: 10.1242/dev.201115 (PMC10110497; doi:10.1242/dev.201115)
Supplement: Supplementary information [file develop-150-201115-s1.pdf]

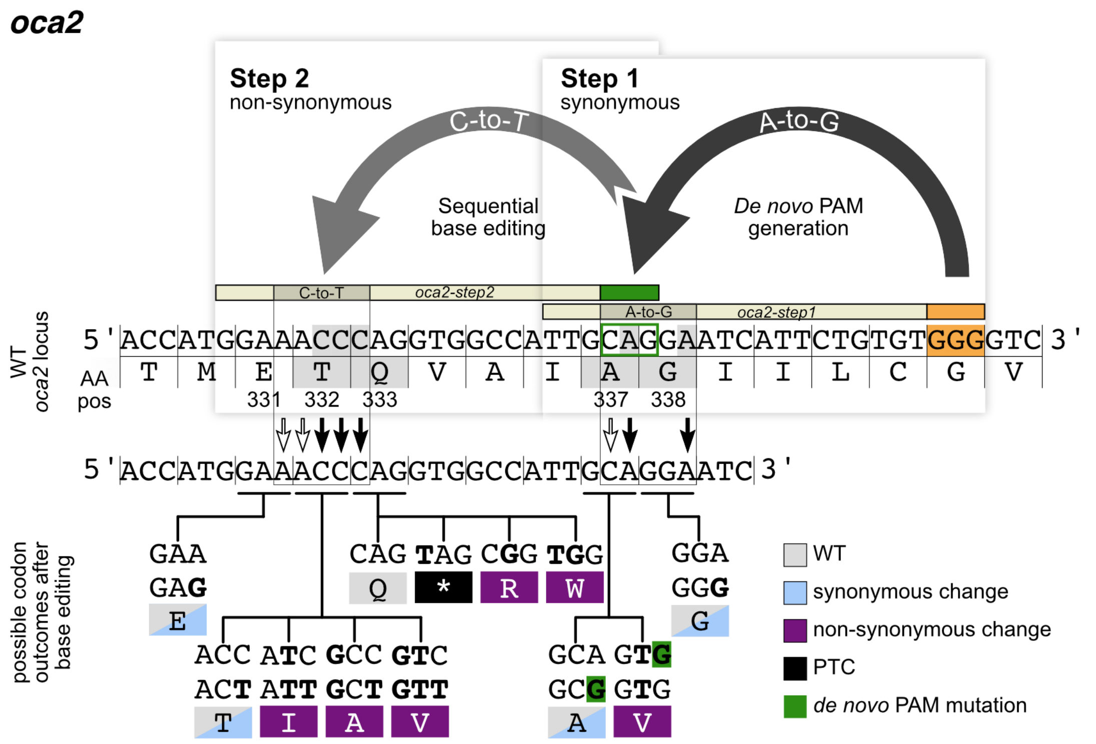

Fig. S1. Range of possible codon outcomes after base editing in *oca2* via inception

Expected range of all potential translated codon outcomes after inception editing using the ABE8e and ancBE4max base editors at the *oca2* locus. The anticipated *de novo* PAM (green outlined box) introduced by *oca2-step1* guide RNA, T332I and Q333* edits introduced by *oca2-step2* guide RNA are indicated by black arrows. Further possible edits are indicated by white arrows. Resulting codon range per amino acid position given. AA pos, amino acid position; canonical PAM (orange); de novo PAM (green); PAM, protospacer adjacent motif; PTC, pre-termination STOP codon; WT, wild-type

Development • Supplementary information

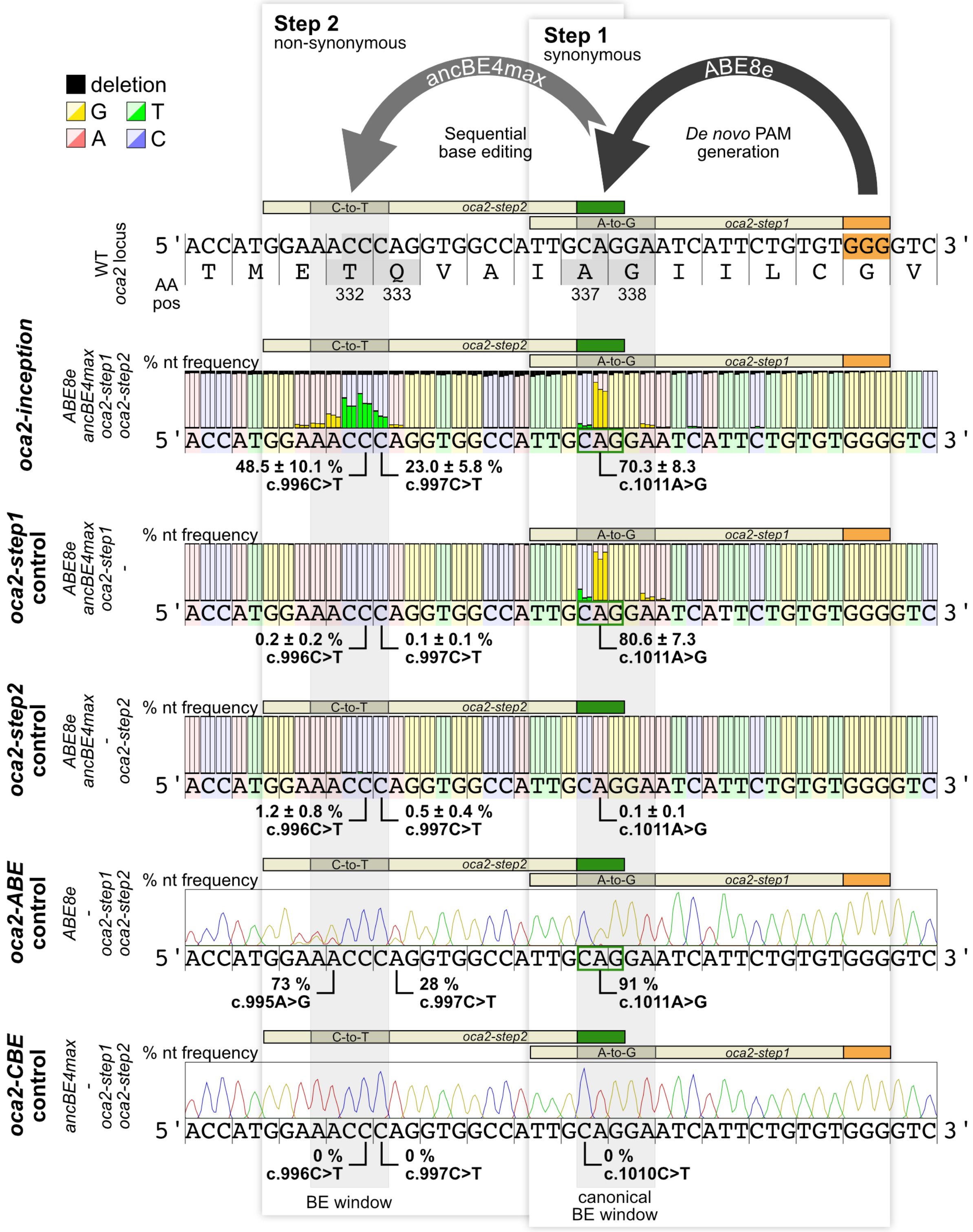
Step 2
non-synonymous
Step 1
synonymous
ancBE4max
ABE8e
Sequential base editing
De novo PAM generation
deletion
G
T
A
C
C-to-T
oca2-step2
A-to-G
oca2-step1
WT oca2 locus
AA pos
5 'ACCATGGAAACCCAGGTGGCCATTGCAGGAATCATTCTGTGTGGGGTC 3 '
T M E T Q V A I A G I I L C G V
332 333 337 338
oca2-inception
ABE8e ancBE4max oca2-step1 oca2-step2
% nt frequency
48.5 ± 10.1 % c.996C>T
23.0 ± 5.8 % c.997C>T
70.3 ± 8.3 c.1011A>G
oca2-step1 control
0.2 ± 0.2 % c.996C>T
0.1 ± 0.1 % c.997C>T
80.6 ± 7.3 c.1011A>G
oca2-step2 control
1.2 ± 0.8 % c.996C>T
0.5 ± 0.4 % c.997C>T
0.1 ± 0.1 c.1011A>G
oca2-ABE control
73 % c.995A>G
28 % c.997C>T
91 % c.1011A>G
oca2-CBE control
0 % c.996C>T
0 % c.997C>T
0 % c.1010C>T
BE window
canonical BE window

Fig. S2. Sequencing results of *oca2* inception and control editants

Illumina amplicon sequencing analyses of *oca2*-editants (three replicates each) injected with the respective injection mixes: *oca2*-inception (3 pools of 8 editants; 246045 reads total), *oca2-step1* control (3 pools of 1 to 8 editants, 82182 reads total) and *oca2-step2* control (3 pools of 8 editants, 124571 reads total). Barplot representation of nucleotide frequency of the three replicates per nucleotide position. Highlighted nucleotide changes represented as mean ± standard deviation.

Chromatograms of *oca2-ABE* (5 editants) and *oca2-CBE* controls (8 editants) resulting from Sanger sequencing with indicated nucleotide changes derived from EditR (Kluesner et al., 2018) analysis.

AA pos, amino acid position; canonical PAM (orange); de novo PAM (green); nt, nucleotide; PTC, pre-termination STOP codon; WT, wild-type

Development • Supplementary information

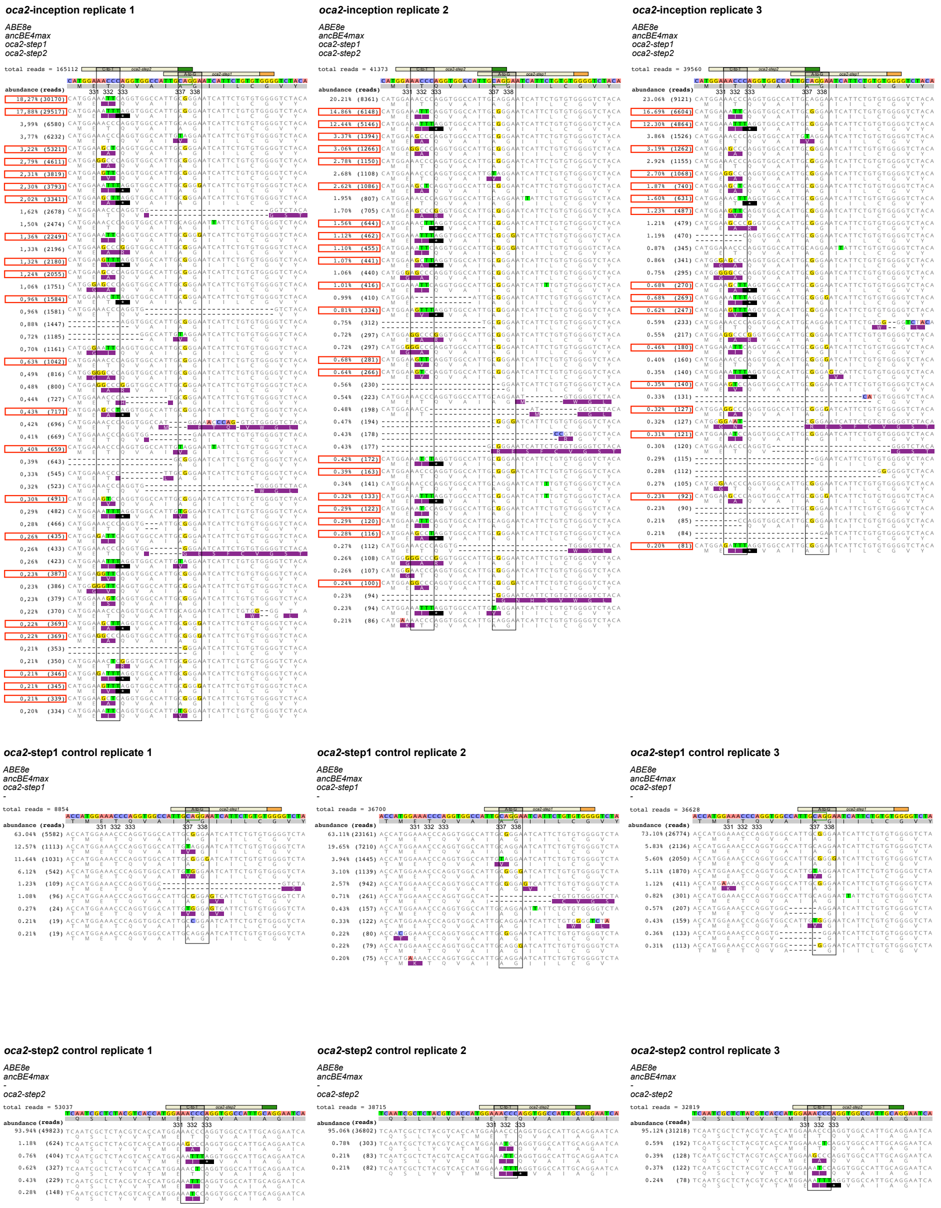
oca2-inception replicate 1
ABE8e
ancBE4max
oca2-step1
oca2-step2
oca2-inception replicate 2
ABE8e
ancBE4max
oca2-step1
oca2-step2
oca2-inception replicate 3
ABE8e
ancBE4max
oca2-step1
oca2-step2
oca2-step1 control replicate 1
ABE8e
ancBE4max
oca2-step1
oca2-step1 control replicate 2
ABE8e
ancBE4max
oca2-step1
oca2-step1 control replicate 3
ABE8e
ancBE4max
oca2-step1
oca2-step2 control replicate 1
ABE8e
ancBE4max
oca2-step2
oca2-step2 control replicate 2
ABE8e
ancBE4max
oca2-step2
oca2-step2 control replicate 3
ABE8e
ancBE4max
oca2-step2

Fig. S3. Allele frequency table and translation of aligned targeted Illumina amplicon sequencing reads of *oca2* inception and control editants

Canonical (step 1) and inception (step 2) guide RNA target sites indicated. Analysis based on the Allele frequency table output files derived from CRISPResso2 tool (Clement et al., 2019), cut off at 0.2 % read abundance per replicate. Alleles sorted by frequency (reads in parentheses). Alignment differences to wild-type reference indicated by color: adenine, red; guanine, yellow; thymidine, green; cytosine, blue; -, deletions. Injection mix components and replicate numbers provided.

Anticipated alleles, red outlined box; base editing window, black outlined box; canonical PAM (orange); *de novo* PAM (green); non-synonymous codon changes, purple box; pre-termination STOP codon, black box

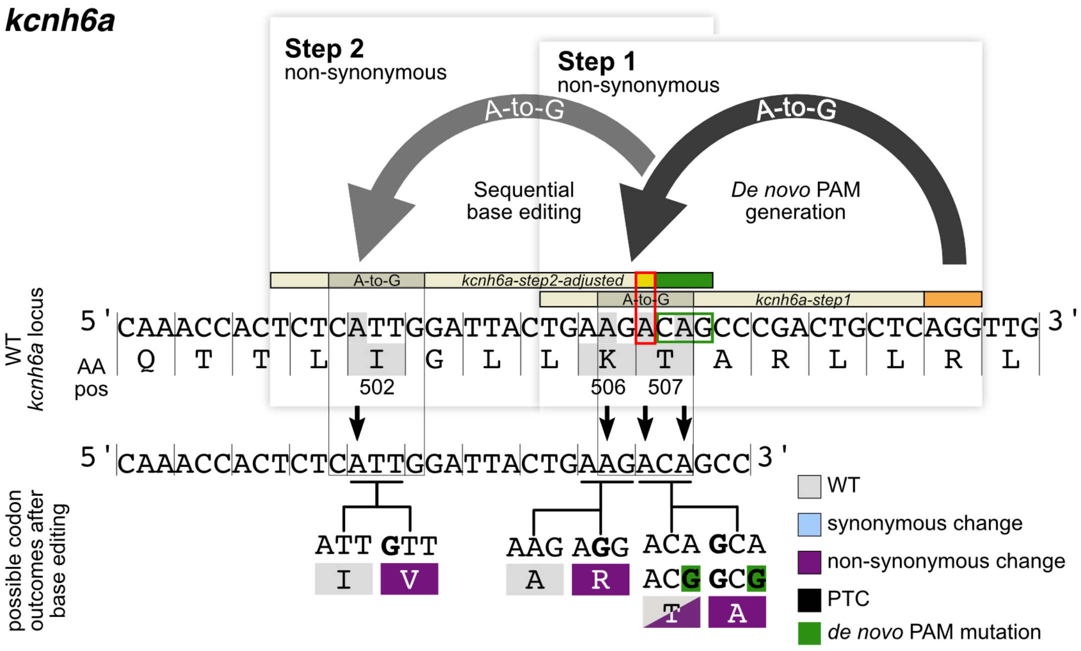

Fig. S4. Range of possible codon outcomes after base editing in *kcnh6a* via inception

Expected range of all potential translated codon outcomes after inception editing using the ABE8e base editor at the *kcnh6a* locus. The anticipated *de novo* PAM (green outlined box) introduced by *kcnh6a-step1* guide RNA and K506R/T507A edits as well as the *kcnh6a-step2-adjusted* guide RNA induced I502V mutations are indicated by black arrows. Resulting codon range per amino acid position given.

AA pos, amino acid position; canonical PAM (orange); de novo PAM (green); PAM, protospacer adjacent motif; WT, wild-type

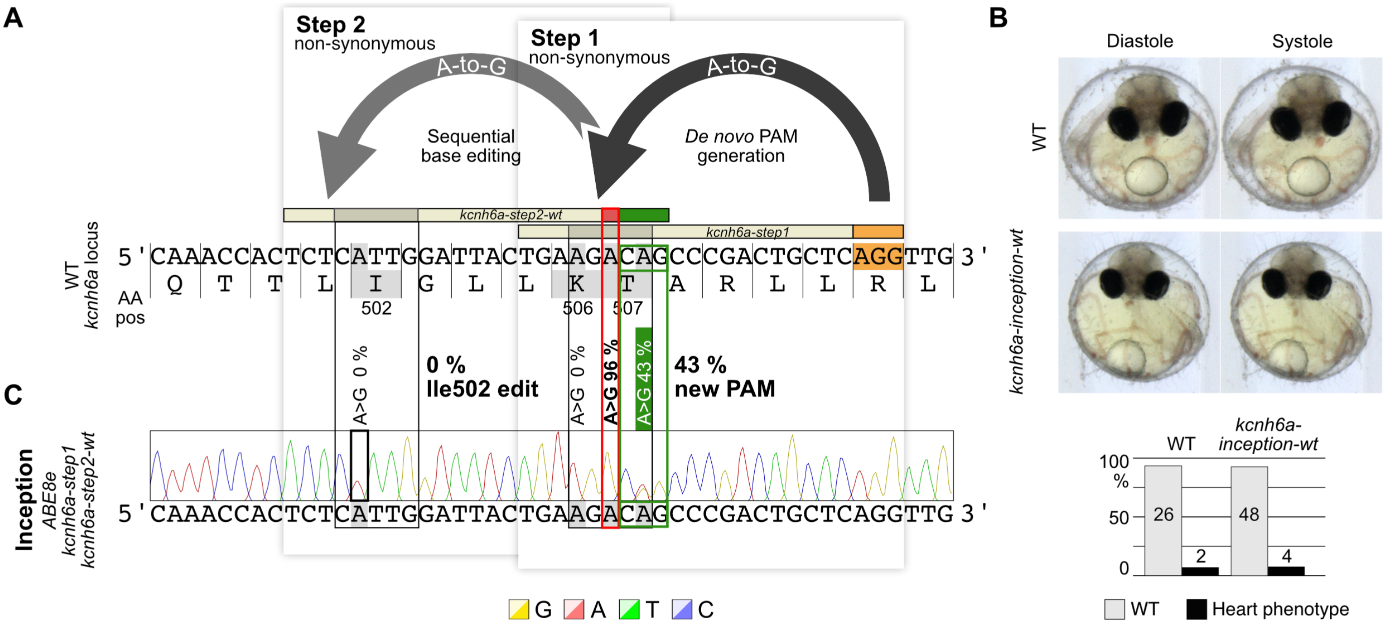

Fig. S5. No inception editing events using wild-type *kcnh6a-step2* guide RNA

(A) Inception mix containing the *kcnh6a-step2-wt* guide RNA fails to edit p.I502 despite the efficient *de novo* PAM generation validated via Sanger sequencing and EditR (Kluesner et al., 2018). The 96 % c.1519A>G editing is in position 1 of the *kcnh6a-step2-wt* target-site preventing the second editing event. (B) At 4 days post fertilization the two-chambered embryonic heart shows Diastole/Systole periodicity. Phenotypically the inception editants were indifferent from wild-type (WT) controls.

AA pos, amino acid position; canonical PAM (orange); de novo PAM (green); red box, sequence difference to edited locus in *kcnh6a-step2-wt* guide RNA

Development • Supplementary information

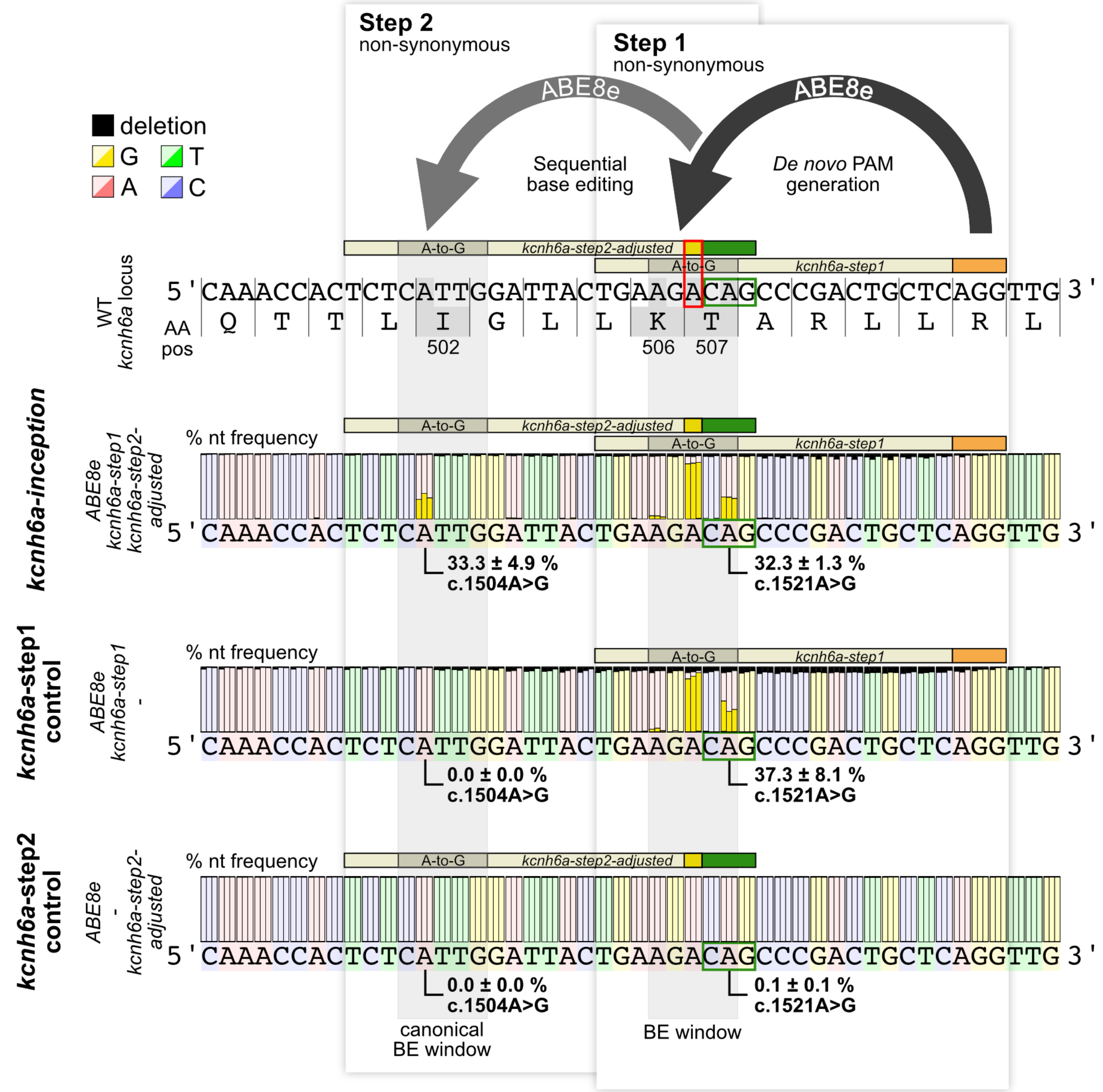

Fig. S6. Sequencing results of kcnh6a inception and control editants Illumina amplicon sequencing analyses of *kcnh6a*-editants (three replicates each) injected with the respective injection mixes: *kcnh6a*-inception (3 pools of 5-10 editants, 164108 reads total), *kcnh6a-step1* control (3 pools of 5 editants, 103384 reads total) and *kcnh6a-step2* control (3 pools of 5, 91445 reads total). Barplot representation of nucleotide frequency of the three replicates per nucleotide position. Highlighted nucleotide changes represented as mean ± standard deviation.

AA pos, amino acid position; canonical PAM (orange); de novo PAM (green); nt, nucleotide; PTC, pre-termination STOP codon; WT, wild-type

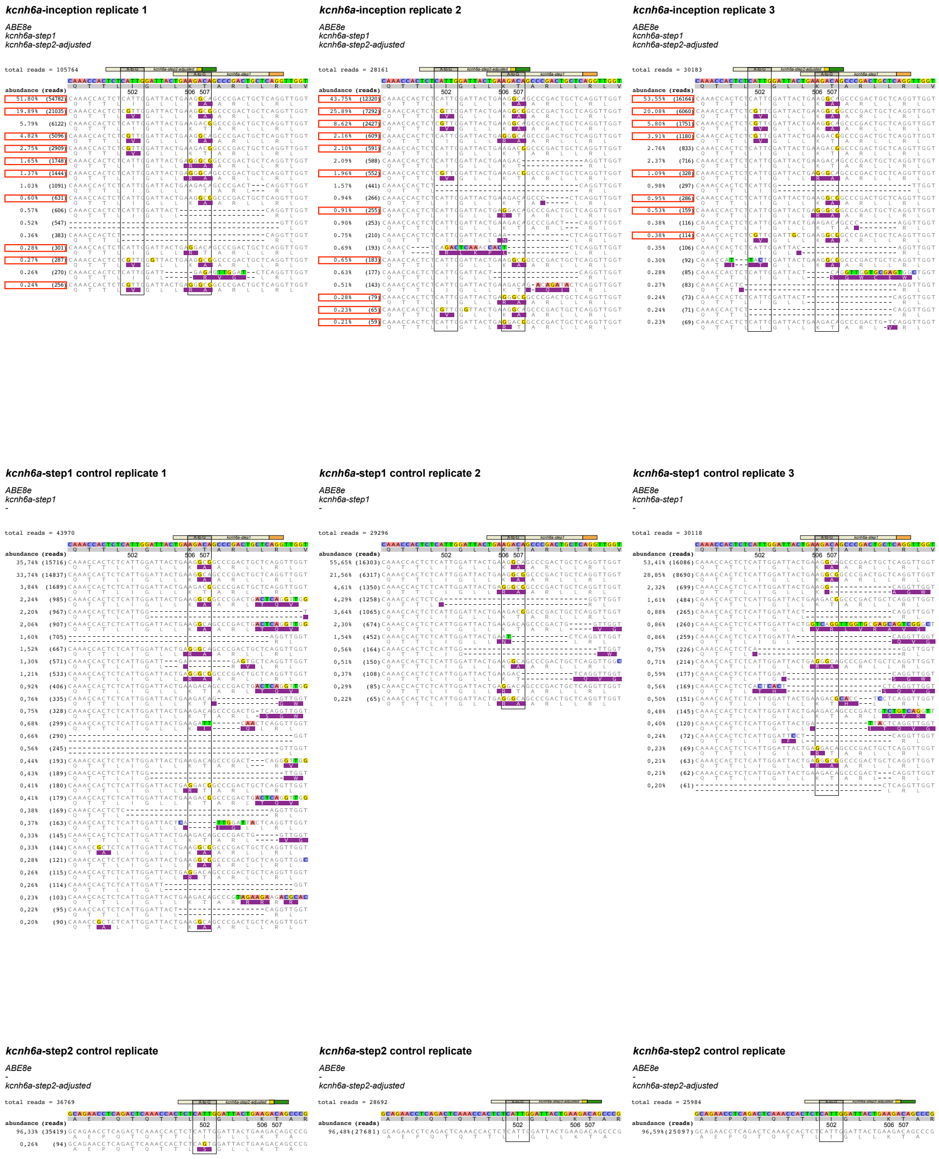

Fig. S7. Allele frequency table and translation of aligned targeted Illumina amplicon sequencing reads of *kcnh6a* inception and control editants

Canonical (step 1) and inception (step 2) guide RNA target sites indicated. Analysis based on the Allele frequency table output files derived from CRISPResso2 tool (Clement et al., 2019), cut off at 0.2 % read abundance per replicate. Alleles sorted by frequency (reads in parentheses). Alignment differences to wild-type reference indicated by color: adenine, red; guanine, yellow; thymidine, green; cytosine, blue; -, deletions. Injection mix components and replicate numbers provided.

Anticipated alleles, red outlined box; base editing window, black outlined box; canonical PAM (orange); *de novo* PAM (green); non-synonymous codon changes, purple box

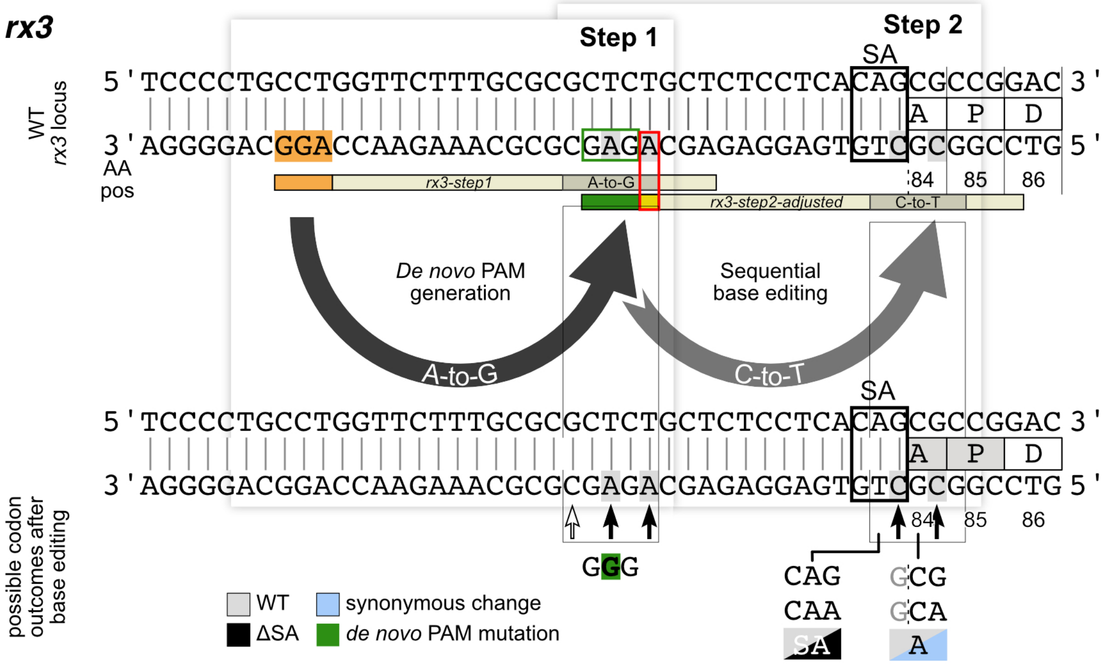

Fig. S8. Range of possible codon outcomes after base editing in *rx3* via inception

Expected range of all potential translated codon outcomes after inception editing using the ABE8e base editor at the *rx3* locus. The anticipated *de novo* PAM (green outlined box) introduced by *rx3-step1* guide RNA and splice acceptor edits (ΔSA) as well as the *rx3-step2-adjusted* guide RNA induced A84A mutations are indicated by black arrows. Resulting codon range per amino acid position given. Further possible edits are indicated by white arrows.

AA pos, amino acid position; canonical PAM (orange); de novo PAM (green); PAM, protospacer adjacent motif; SA, splice acceptor; WT, wild-type

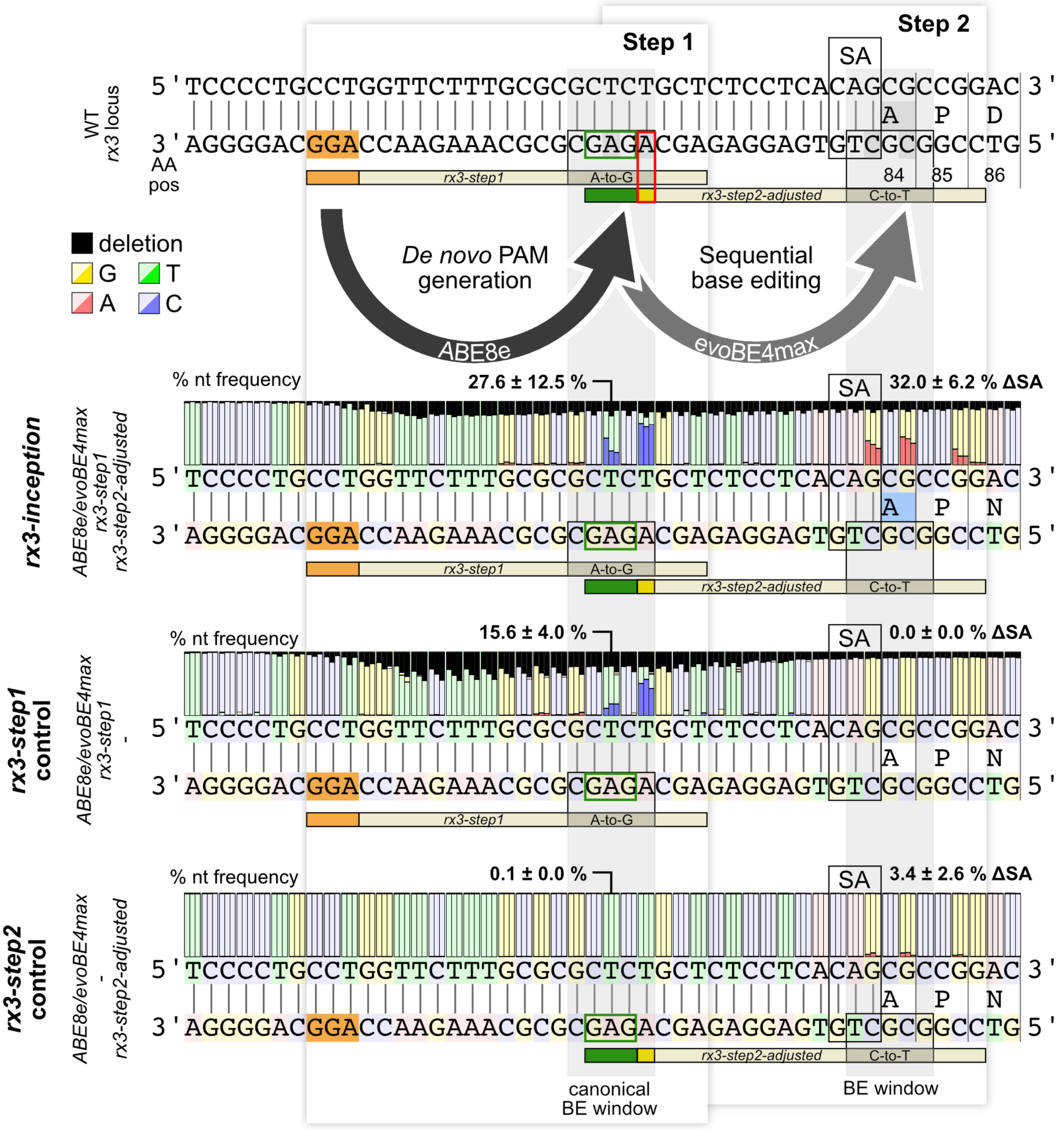

Development • Supplementary information

Fig. S9. Sequencing results of rx3 inception and control editants

Illumina amplicon sequencing analyses of *rx3*-editants (three replicates each) injected with the respective injection mixes: *rx3-inception* (3 pools of 5-9 editants, 136642 reads total), *rx3-step1* control (3 pools of 5 editants, 97976 reads total) and *rx3-step2* control (3 pools of 5 editants, 82283 reads total). Barplot representation of nucleotide frequency of the three replicates per nucleotide position. Highlighted nucleotide changes represented as mean ± standard deviation.
AA pos, amino acid position; canonical PAM (orange); de novo PAM (green); nt, nucleotide; PTC, pre-termination STOP codon; SA, splice acceptor site; WT, wild-type

Development • Supplementary information

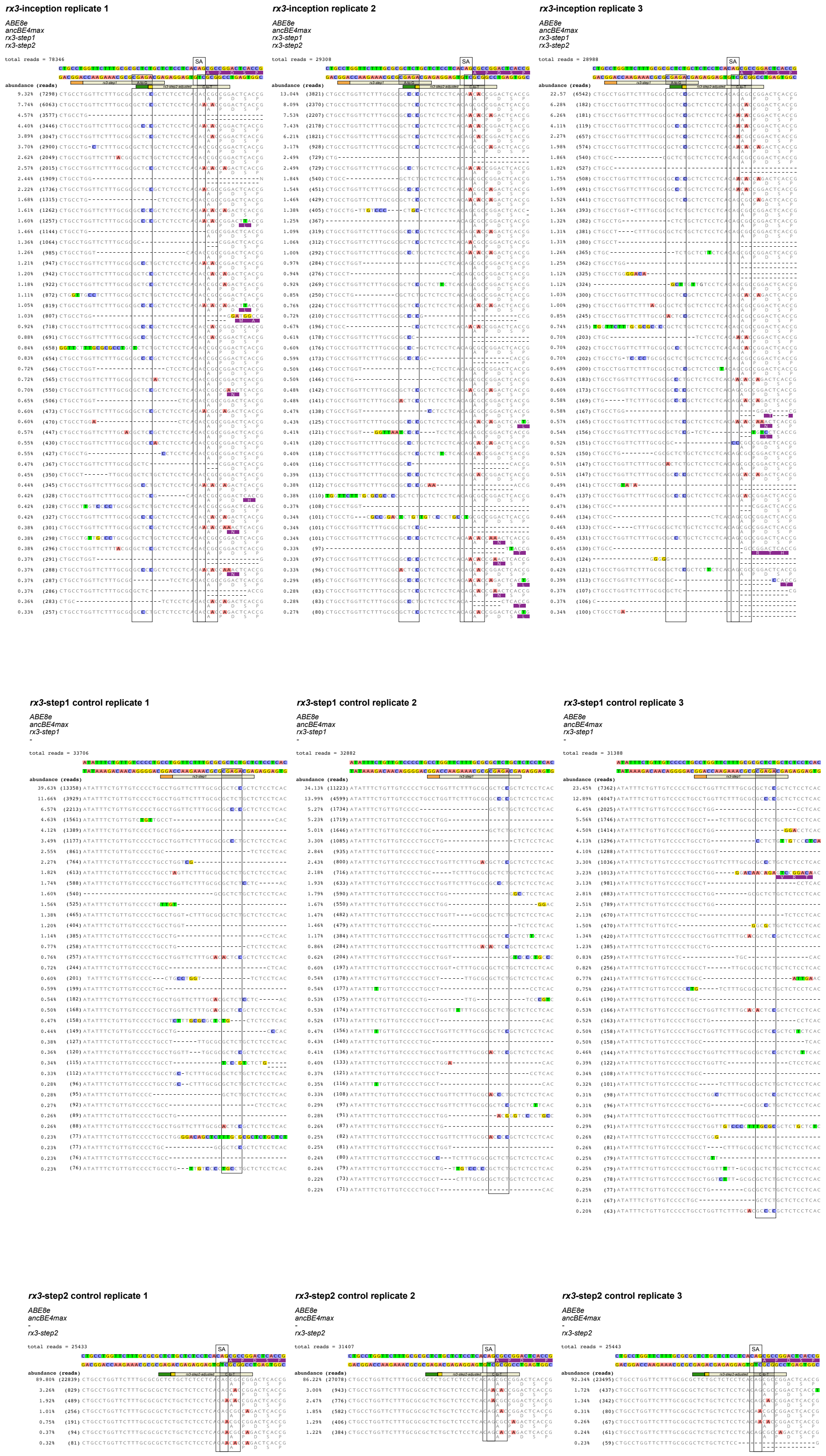

Development • Supplementary information

Fig. S10. Allele frequency table and translation of aligned targeted Illumina amplicon sequencing reads of *rx3* inception and control editants

Canonical (step 1) and inception (step 2) guide RNA target sites indicated. Analysis based on the Allele frequency table output files derived from CRISPResso2 tool (Clement et al., 2019), cut off at 0.2 % read abundance per replicate. Alleles sorted by frequency (reads in parentheses). Alignment differences to wild-type reference indicated by color: adenine, red; guanine, yellow; thymidine, green; cytosine, blue; -, deletions. Injection mix components and replicate numbers provided.

Base editing window, black outlined box; canonical PAM (orange); *de novo* PAM (green); non-synonymous codon changes, purple box; SA, splice acceptor site

Development • Supplementary information

Table S1. Raw data of base editor target site abundances in the top ten studied human genes and the orthologs in mouse, zebrafish, medaka and drosophila

Click here to download Table S1

Table S2. Overview of injection mixes and analyzed embryos across all replicates and targeted loci

GFP, green fluorescent protein; WT, wild-type

Click here to download Table S2

Table S3. Guide RNAs used in this study

Guide RNA name	Target Site [PAM] 5’-3’	Reference
oca2-step1 sgRNA	TTGCAGGAATCATTCTGTGT[GGG]	(Hammouda et al., 2019)
oca2-step2 sgRNA	GGAAACCCAGGTGGCCATTG[CAG]	this paper
kcnh6a-step1 sgRNA	TGAAGACAGCCCGACTGCTC[AGG]	(Cornean et al., 2022)
kcnh6a-step2-wt crRNA	TCTCATTGGATTACTGAAGA[CAG]	this paper
kcnh6a-step2-adjusted crRNA	TCTCATTGGATTACTGAAGg[CAG]	this paper
rx3-step1 sgRNA	AGCAGAGCGCGCAAAGAACC[AGG]	(Zilova et al., 2021)
rx3-step2-adjusted crRNA	CCGGCGCTGTGAGGAGAGCg[GAG]	this paper

Table S4. Primers with partial Illumina adapter sequences (underscored) used in this study

Primer name	Adapter Sequence, Primer sequence 5’-3’
oca2_F	ACACTCTTTCCCTACACGACGCTCTTCCGATCTCGTTAGAGTGGTATGGAGAACTGT
oca2_R	GACTGGAGTTCAGACGTGTGCTCTTCCGATCTATGGTCCTCACATCAGCAGC
kcnh6a_F	ACACTCTTTCCCTACACGACGCTCTTCCGATCTAGTTTGCTGTGTACCTCCAGTT
kcnh6a_R	GACTGGAGTTCAGACGTGTGCTCTTCCGATCTATCTTCATACCGCCCACACG
rx3_F	ACACTCTTTCCCTACACGACGCTCTTCCGATCTATGCAAACCAAGAAAGCGCC
rx3_R	GACTGGAGTTCAGACGTGTGCTCTTCCGATCTTGGGATTTCTCAAAGGCCCG

Table S5. Primers used in this study

Primer name	Sequence 5’-3’
oca2_F	GTTAAAACAGTTTCTTAAAAAGAACAGGA
oca2_R	AGCAGAAGAAATGACTCAACATTTTG
kcnh6a_F	GCTTTGCAAGGTATAGAGCACAG
kcnh6a_R	AACGTTGCCAAAACCCACAC

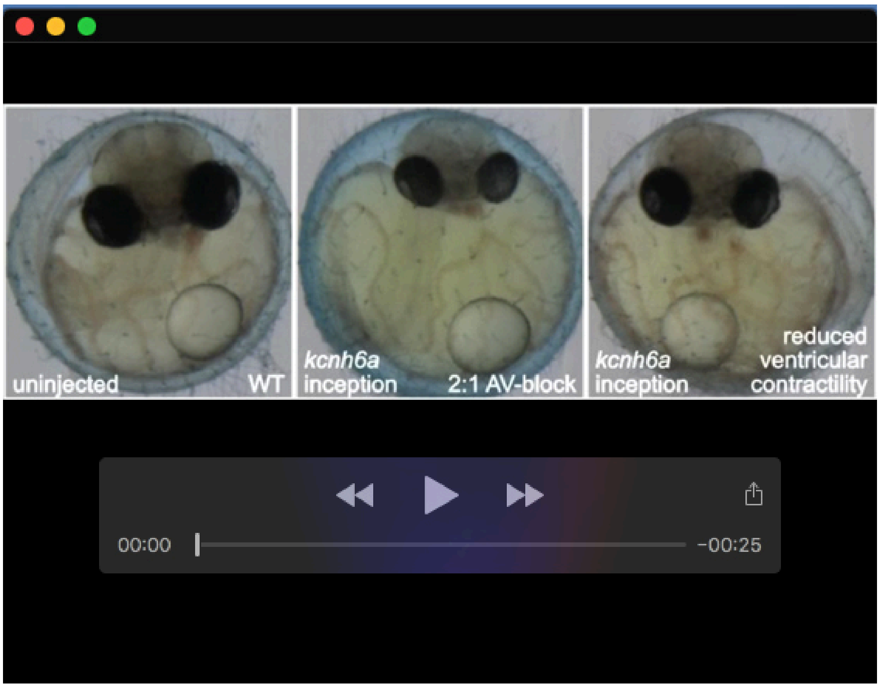

Movie 1. Exemplary heart phenotypes of *kcnh6a*-inception editants
10 second movies of heart beat and heart morphology analysis at stage 35 of wild-type (WT, left) and *kcnh6a*-inception injected embryos. Representative phenotypes cover 2:1 atrioventricular block (middle) and reduced ventricular contractility (right). A, atrium; V, ventricle; movie plays at 10 frames per second (real time)

Supplementary References

Clement, K., Rees, H., Canver, M. C., Gehrke, J. M., Farouni, R., Hsu, J. Y., Cole, M. A., Liu, D. R., Joung, J. K., Bauer, D. E., et al. (2019). CRISPResso2 provides accurate and rapid genome editing sequence analysis. *Nat Biotechnol* **37**, 224-226.

Cornean, A., Gierten, J., Welz, B., Mateo, J. L., Thumberger, T. and Wittbrodt, J. (2022). Precise in vivo functional analysis of DNA variants with base editing using ACEofBASEs target prediction. *Elife* **11**.

Hammouda, O. T., Bottger, F., Wittbrodt, J. and Thumberger, T. (2019). Swift Large-scale Examination of Directed Genome Editing. *PLoS One* **14**, e0213317.

Kluesner, M. G., Nedveck, D. A., Lahr, W. S., Garbe, J. R., Abrahante, J. E., Webber, B. R. and Moriarity, B. S. (2018). EditR: A Method to Quantify Base Editing from Sanger Sequencing. *CRISPR J* **1**, 239-250.

Zilova, L., Weinhardt, V., Tavhelidse, T., Schlagheck, C., Thumberger, T. and Wittbrodt, J. (2021). Fish primary embryonic pluripotent cells assemble into retinal tissue mirroring in vivo early eye development. *Elife* **10**.

Development • Supplementary information
