# Supplementary material for: De novo PAM generation to reach initially inaccessible target sites for base editing
Source: Development. 2023 Jan 23;150(2):dev201115. doi: 10.1242/dev.201115 (PMC10110497; doi:10.1242/dev.201115)
Supplement: Supplementary file 1 [file develop-150-201115_review_history.pdf]

Development

De novo PAM generation to reach initially inaccessible target sites for base editing

Kaisa Pakari, Joachim Wittbrodt and Thomas Thumberger

DOI: 10.1242/dev.201115

Editor: Steve Wilson

Review timeline

Original submission:	7 July 2022
Editorial decision:	12 August 2022
First revision received:	15 November 2022
Editorial decision:	12 December 2022
Second revision received:	15 December 2022
Accepted:	16 December 2022

Original submission

First decision letter

MS ID#: DEVELOP/2022/201115

MS TITLE: The inceptionist's guide to base editing - de novo PAM generation to reach initially inaccessible target-sites

AUTHORS: Kaisa Pakari, Joachim Wittbrodt, and Thomas Thumberger

I have now received all the referees' reports on the above manuscript, and have reached a decision. The referees' comments are appended below, or you can access them online: please go to BenchPress and click on the 'Manuscripts with Decisions' queue in the Author Area.

As you will see, the referees express considerable interest in your work, but have some criticisms and suggestions for improving your manuscript. If you are able to revise the manuscript along the lines suggested, I will be happy receive a revised version of the manuscript. Please also note that Development will normally permit only one round of major revision. If it would be helpful, you are welcome to contact us to discuss your revision in greater detail. Please send us a point-by-point response indicating your plans for addressing the referee's comments, and we will look over this and provide further guidance.

Please attend to all of the reviewers' comments and ensure that you clearly highlight all changes made in the revised manuscript. Please avoid using 'Tracked changes' in Word files as these are lost in PDF conversion. I should be grateful if you would also provide a point-by-point response detailing how you have dealt with the points raised by the reviewers in the 'Response to Reviewers' box. If you do not agree with any of their criticisms or suggestions please explain clearly why this is so.

Reviewer 1

Advance summary and potential significance to field

The manuscript by Pakari, Wittbrodt and Thumberger presents a very exciting idea of extending the repertoire of base editable sites by using adenine base editors to introduce ("incept") new PAM sites. This work is highly significant and, with caveats outlined below, has tremendous potential.

© 2023. Published by The Company of Biologists under the terms of the Creative Commons Attribution License (https://creativecommons.org/licenses/by/4.0/).

Comments for the author

Major comments:

1. As the authors note on lines 149-152, it is very noteworthy that when editing both oca2 and rx3, the adenine base editor used to "incept" the new PAM thoroughly outperformed the cytosine base editor at the first editing site. Yet, at the second ("incepted") editing site in oca2, the cytosine base editor appears to have thoroughly outperformed the adenine base editor. Is it by pure luck, or is there a sensible biological explanation for this observation (say, the adenine base editor folds much faster but is less stable)? How likely is this "higher activity at the intended target site" to hold up across other loci and, importantly, in other species?

2. The "inception efficiency" numbers provided in Figure 2 seem to be arbitrary and meaningless. The number that really matters is the fraction of precise desired double edits. This is especially true considering potential application in humans inferred in the first sentence of Introduction and Figure 1B. Based on Figure S1 that number would be 17.88% for oca2 - which is terrific. For rx3, the majority of first site edits seem to have both As within the editing window mutated to Gs. Likewise, in the "incepted" edit, both Cs within the editing window are likely to be mutated to Ts (not just the one which is a part of the splice site). These observations complicate definition and calculation of "precise double edit", but it has to be done. In addition the observation that a substantial fraction of reads show an additional C-to-T edit right outside the expected editing window needs to be mentioned. Finally, for kcnh6a, all missense alleles represent desired outcomes and therefore frequency of each of the single/double/triple mutants (T507A, K506R, T507A/K506R T507A/I502V, K506R/I502V, T507A/K506R/I502V) should be represented in the main figure.

Minor comments:

1. Figure 2I contains an error in the "Edited allele" line: AGG should be the codon for arginine.

<u>Reviewer 2</u>

Advance summary and potential significance to field

CRISPR systems have restricted sequence access due to their traditional PAM sequences. This paper describes a clever, dual CRISPR base editing strategy to add a new PAM nearby an existing site to make additional targeted gene alleles.
They use the medaka, but this approach is likely well-suited for any animal model used in development.

Comments for the author

This paper describes a clever and very elegant approach to enhance CRISPR base editing approaches through a sequential approach of enhanced PAM sequence generation followed by base editing. They use the medaka as an animal model. The data on the included loci show strong success rates, making PTC, altered splice sites and hyomorphic alleles. The authors themselves seem surprised at how effective this approach is.

As presented, the study is generally well-devised and convincing of strong somatic gene editing functionality of this combinatorial approach. Phenotypic and molecular data strongly correlate. Control injections of individual editors show little or no phenotypes.

The major issue that is unexplored is the true specificity of this overall approach. Base editors DO cause deletions at a finite rate due to the nicks induced to help with preferential DNA strand repair. There are several ways to address this question - 1) deep-dive into the somatic cell edits. 2) explore animals after germline passage. A failure to recover germline chromosomes would be a clear dataset that suggests there are more changes to the genome than anticipated/noted by the PCR testing they deployed.

© 2023. Published by The Company of Biologists under the terms of the Creative Commons Attribution License (https://creativecommons.org/licenses/by/4.0/).

Minor issues:
1. How many independent replicates are presented for each assay? This is not clear from the abbreviated format.
2. There are too many significant digits presented for nearly all of the presented data. '26.1%' - what are the error estimates? Most likely in the 5-10% range. The range of outcomes should be presented.
3. I'm not a fan of new jargon, such as the phrase 'inceptionist' in the title. This did not enhance scientific communication in my personal view.

Reviewer 3

Advance summary and potential significance to field

A major limitation to generating mutations that model human disease-associated SNPs in cells or model organisms using base editors designed around Cas9 is the requirement for a PAM sequence 13-17 nt downstream of the desired edit site. A number of modifications to the base editor enzyme have been described that alter or relax the strict PAM requirement of Cas9, however none of these are perfect and there is a constant need for alternative strategies for precise genome engineering. This manuscript describes a new strategy, "Inception", that uses base editors to first generate a new PAM site, and then to use that site to make the desired edit, in a one-pot (actually, one embryo) reaction. The net effect is increase the number of nucleotides accessible for editing by a factor of about 1.6: now edits can additionally be made in a window 27-36 upstream of a canonical PAM site.

Using genes with known phenotypes in Medaka fish as an experimental paradigm, and injecting sgRNAs and base editor mRNAs into embryos at the one-cell stage, the authors demonstrate successful examples of sequential editing to generate a novel NGG PAM followed by editing to generate desired mutations. While the authors don't attempt to generate germline alleles for any of these edits, at this point there is sufficient proof in the literature that mosaic mutations can be passed through the germline that there is little doubt that these will too.

Comments for the author

The work represents a significant advance and merits publication as a technical report in Development, however the text is very brief and both the text and the figures contain many inconsistencies and omissions that make it difficult to follow and understand.

1) The authors should state more clearly at the outset in the main text (not just the methods) what their percentages refer to. For example, line 145: does 79.7% refer to the percentage of sequenced PCR amplicons containing the 1011A>G mutation, or the percentage of larvae in which that mutation was detected? If it is the former, then how many amplicons from how many larvae were sequenced? Were the larvae that were sequenced chosen randomly, or were they the most phenotypically affected?

2) In line 146, does 26.1% refer to the percentage of 1011A>G containing amplicons that also have the Gln333* mutation, or the percentage of all amplicons containing the Gln333* mutation?

3) Fig. 2 includes a statement of "Inception efficiency" that is not explained until the very end of the text (line 196). Since the ultimate goal of the approach is to generate a specific targeted mutation (e.g. Q333* for oca2; splice acceptor loss for rx3) without generating non-synonomous mutations at the new PAM site, the "inception efficiency" percentage stated in Fig. 2 should be the efficiency with which this precise goal was accomplished, i.e. the percentage of amplicons with this mutation and no other non-synonomous mutation. This does not appear currently to be the case, if, for example 26% of oca2 amplicons have a PTC, the inception efficiency can't be 75.5%.

4) Since the new PAM site generated in oca2 is one nucleotide upstream of an existing PAM site (AGG), the authors have missed the opportunity to compare the efficiency of a conventional one-step C-to-T base editing event to the efficiency of Inception.

© 2023. Published by The Company of Biologists under the terms of the Creative Commons Attribution License (https://creativecommons.org/licenses/by/4.0/).

5) The authors comment (line 150-151) that the two base editors appear to show higher activity at their respective intended target sites. This is a completely mysterious finding that should be explained, however the only controls that are provided all involve injecting both base editors (A-to-G and C-to-T) with one or the other sgRNA. Controls should be performed in which both sgRNAs are injected with single base editors.

6) The text and Table S2 refer to “inception sgRNAs” (in the case of oca2) and “inception-adjusted sgRNAs” (in the case of rx3 and kcnh6a). What is the difference between these? Why is the experimental condition for oca2 and rx3 “inception” and the experimental condition for kcnh6a “inception-adjusted”? Also, what is the difference between “newPAM control” (Table S1 and Table S2 for oca2) and “canonical PAM control” for Table S2 rx3 and kcnh6a? If these are real differences they need to be carefully explained, but if they are actually the same, they should be named the same way to spare your readers a great deal of confusion.

7) In general, the section of the main text describing the kcnh6a approach is very confusing. What is the “inception-original cr/trRNA” mentioned at the bottom of Table S2 and in Fig. S3? Which data does it correspond to in Table S3? In the methods, the kcnh6a-inception-original crRNA and the kcnh6a-inception adjusted crRNA appear to have the identical sequence: TCTCATTGGATTACTGAAGg[CAG].

8) In table S2, information should be provided on the number of oca2-edited larvae that had with oca2 phenotypes (mosaic eyes).

9) The term “unedited codons” which appears in Table S3 should be mentioned in the main text and it should be explained why edited and unedited codon percentages do not add up to 100%

10) Table S3 section on rx3 editing is very confusing. It contains no information on the efficiency of de-novo PAM generation frequency, although it is stated as 42% in the text and Fig. 2, and it contains no information on the mutation frequency at the target splice acceptor site, stated as 38% in the text and Fig. 2. The methods states that genomic DNA, not cDNA was extracted from injected embryos, so it’s not clear why intron sequence was not captured. Intron sequence including both edit sites should be included for all three conditions (inception, newPAM control and rx3 inception control).

Minor comments:

1) This reviewer does not like the term “editants” which, unlike “morphant” and “CRISPant” is too vague to be a useful short-form.

2) The term “literally” (line 187) should be removed. Scientific reporting should always be literal. Paradoxically, in this case “literally devoid” is meant to mean “almost but not quite entirely devoid…” (2/57 embryos had heart phenotypes).

3) The title of the paper (“The inceptionist’s guide...”) makes it sound like a review rather than an original work. Recommend changing to “de-novo PAM generation to reach initially inaccessible target sites using base editing”

First revision

Author response to reviewers' comments

Reviewer 1 Advance Summary and Potential Significance to Field...

The manuscript by Pakari, Wittbrodt and Thumberger presents a very exciting idea of extending the repertoire of base editable sites by using adenine base editors to introduce ("incept") new PAM sites. This work is highly significant and, with caveats outlined below, has tremendous potential.

© 2023. Published by The Company of Biologists under the terms of the Creative Commons Attribution License (https://creativecommons.org/licenses/by/4.0/).

Reviewer 1 Comments for the Author...

Major comments:

1. As the authors note on lines 149-152, it is very noteworthy that when editing both oca2 and rx3, the adenine base editor used to "incept" the new PAM thoroughly outperformed the cytosine base editor at the first editing site. Yet, at the second ("incepted") editing site in oca2, the cytosine base editor appears to have thoroughly outperformed the adenine base editor. Is it by pure luck, or is there a sensible biological explanation for this observation (say, the adenine base editor folds much faster but is less stable)? How likely is this "higher activity at the intended target site" to hold up across other loci and, importantly, in other species?
We thank the reviewer for critical reading and pointing us to the missed explanation. Base editing efficiency is strongly context dependent, i.e. the nucleotide composition of the target site has a strong impact on individual nucleotide editing efficiencies. More precisely, the dinucleotide context, i.e. the influence of the preceding nucleotide on the editing efficiency can explain the observed differences in editing of both base editors at each target site. In the case of the ABE8e, a preceding adenine as present in the E331/T332 codons (i.e. step 2 in *oca2*) can drastically reduce the editing efficiency as we have shown earlier in Cornean et al., 2022 (eLife, doi: 10.7554/eLife.72124). The same is true for the canonical base editing window (*oca2-step1*) in which the cytosine of codon A337 (GCA) is preceded by a guanine which reduces the efficiency of the ancBE4max (Cornean et al., 2022). Thus, the base editors perform more efficiently on their respective intended target site. We have now included this explanation in the main text. The same AA dinucleotide influence on the ABE8e efficiency can also be seen in the canonical base editing window of *kcnh6a* – here c.1517A>G is poorly edited (3.4%±1%), although contained within the base editing window. We also discuss this in the main text.
In the case of *rx3*, the influence on editing by the preceding G in the “GC” dinucleotide is more variable, and apparently very negative in this particular case (*rx3-step1*) – see again Cornean et al., 2022.

2. The "inception efficiency" numbers provided in Figure 2 seem to be arbitrary and meaningless.
We agree with the reviewer and have removed the “inception efficiency” calculations.

The number that really matters is the fraction of precise desired double edits. This is especially true considering potential application in humans inferred in the first sentence of Introduction and Figure 1B.
We are grateful to the reviewer for making us realize that we focused too much on a very special case (potential application in humans) in the introduction. We now emphasize more on the power of our inception approach, which allows for the generation of animal disease models, hypomorphic allele studies and the efficient generation of gene knock-outs. Further we redefined what we mean by precision and desired edits: the nucleotides in the base editing window define the possible codon outcomes. We now have included Supplementary figures defining this base editor range (S1, S4, S8) per locus. In the case of *oca2*, in which we anticipated to introduce a pre-termination STOP codon (CAG>TAG), the leading codon of Threonine 332 (ACC) is contained within the same base editing window and thus is likely to be edited in parallel. From our previous study (Cornean et al., 2022) we already found T332A editing to cause mild loss-of-pigmentation. Thus, loss of function can be reached by both, T332 as well as Q333* mutation, which made us rephrase our goal to demonstrate a “loss-of-function regimes by introducing non-synonymous codon changes including a pre-termination STOP codon (PTC) in an open reading frame” of oca2.
In summary we now show that 48.4% of edited alleles depict the anticipated loss-of- function variations in *oca2* (Fig 2D).

Based on Figure S1 that number would be 17.88% for oca2 - which is terrific.
We agree with the reviewer and following further suggestions we have reworked display of these numbers (Fig2C and updated Figure S2 - see below).

For rx3, the majority of first site edits seem to have both As within the editing window mutated to Gs. Likewise, in the "incepted" edit, both Cs within the editing window are likely to be mutated to Ts (not just the one which is a part of the splice site). These observations complicate definition and calculation of "precise double edit", but it has to be done.

© 2023. Published by The Company of Biologists under the terms of the Creative Commons Attribution License (https://creativecommons.org/licenses/by/4.0/).

From the comment of the reviewer, we realized that we did not fully succeed in conveying our message. We want to disrupt gene function either by introduction of a PTC or by manipulation of the splice acceptor site and as such further codon changes are negligible. Across all *rx3* replicates we now have calculated the efficiency of new PAM introduction (27.6 % ± 12.5 %) and the subsequent mutation of the CAG splice acceptor site to CAA (32.0 % ± 6.1 % (now included in new Fig. 4C).

In addition, the observation that a substantial fraction of reads show an additional C-to-T edit right outside the expected editing window needs to be mentioned.
The base editing window comprising 5 nucleotides demarcates the “general base editing window”. Depending on the target sequence and choice of base editor, this window can be broader than 5 nucleotides (see Cornean et al., 2022). We now address this point in the main text as well.

Finally, for kcnh6a, all missense alleles represent desired outcomes, and therefore frequency of each of the single/double/triple mutants (T507A, K506R, T507A/K506R, T507A/I502V, K506R/I502V, T507A/K506R/I502V) should be represented in the main figure.
We are grateful to the reviewer, to request an in-depth analysis of the Illumina reads. We now have extended the old Figure S1 with the matching allele-based codon translations to calculate the requested anticipated edited alleles (new Fig. S7). We display the abundances of the anticipated outcomes in Figure 3D. We likewise have extended this analysis for *oca2* (Fig 2D, Fig S3).

Minor comments:

1. Figure 2I contains an error in the "Edited allele" line: AGG should be the codon for arginine.
We thank the reviewer for critical reading, and have corrected the mistake

Reviewer 2 Advance Summary and Potential Significance to Field...

CRISPR systems have restricted sequence access due to their traditional PAM sequences. This paper describes a clever, dual CRISPR base editing strategy to add a new PAM nearby an existing site to make additional targeted gene alleles. They use the medaka, but this approach is likely well-suited for any animal model used in development.

Reviewer 2 Comments for the Author...

This paper describes a clever and very elegant approach to enhance CRISPR base editing approaches through a sequential approach of enhanced PAM sequence generation followed by base editing. They use the medaka as an animal model. The data on the included loci show strong success rates, making PTC, altered splice sites and hyomorphic alleles. The authors themselves seem surprised at how effective this approach is.

As presented, the study is generally well-devised and convincing of strong somatic gene editing functionality of this combinatorial approach. Phenotypic and molecular data strongly correlate. Control injections of individual editors show little or no phenotypes.

The major issue that is unexplored is the true specificity of this overall approach. Base editors DO cause deletions at a finite rate due to the nicks induced to help with preferential DNA strand repair. There are several ways to address this question - 1) deep-dive into the somatic cell edits.
We thank the reviewer for raising this important point. We fully agree with the reviewer that base editors do cause indels, which we had addressed at all targeted loci by Illumina sequencing already in the initially submitted manuscript. We have taken care that this important point is particularly stressed in the revised version and have edited the text accordingly. In addition, we have taken the suggested deep-dive and have introduced replicate analyses using Illumina sequencing to further support our statements.

Strikingly, indel formation is clearly locus dependent as seen at the *rx3* locus with a rate of up to 37%±8. The control experiments clearly showed that the phenotype did not correlate with indel formation but the introduction of specific base edits, c.f. *rx3-step1* control that shows substantial indel formation but low phenotypic prevalence versus *rx3-* inception with less indel formation but strong impairment of eye formation upon splice- acceptor removal (new Figure 4, new

© 2023. Published by The Company of Biologists under the terms of the Creative Commons Attribution License (https://creativecommons.org/licenses/by/4.0/).

Supplementary Figure S10). The scope of our publication is not to optimize the base editors themselves, but to provide a novel way of using them.
Since the control experiments do not show the phenotype rate seen in the inception injections, we conclude that phenotype causing mutations are well on-target.

2) explore animals after germline passage. A failure to recover germline chromosomes would be a clear dataset that suggests there are more changes to the genome than anticipated/noted by the PCR testing they deployed.
We followed the reviewers advise and have chosen to address option 1 (see above) but did not want to leave this important point unaddressed.
We have focused on option 1 since option 2 required more time on the one hand and since we had in part already addressed option 2 in previous analyses.
All loci addressed here reflect "standard" targeted genome editing sites in the lab that were addressed at depth in transient and stable lines. In Cornean et al., 2022 (eLife, doi: 10.7554/eLife.72124) we have shown that specific base edits and the resulting phenotypes are fully correlated in F0 individuals as much as in F1 homozygous animals. While indels occur (as observed in the Illumina analysis detailed above) there was no apparent contribution to the phenotype in the cases addressed by a comparative F0/F1 analysis in line with the relatively low rate of their occurrence. This does not exclude a possible contribution of indels at another locus and needs to be considered in the analysis. We have stated that clearly in the revised version of the manuscript.

Minor issues:
1. How many independent replicates are presented for each assay? This is not clear from the abbreviated format.
We thank the reviewer for pointing this out and we now show Illumina sequencing reads and phenotyping of three independent replicates per locus and injection mixes. The main figures contain mean ± standard deviation of the nucleotide changes, and in Supplementary Figures S2, S6 and S9 all replicates are displayed individually. Numbers of injected embryos per replicate (updated Supplementary Table S2), numbers of embryos pooled for sequencing, Illumina sequencing reads and calculated allele frequency abundances are now unambiguously provided throughout the manuscript.

2. There are too many significant digits presented for nearly all of the presented data. '26.1%' - what are the error estimates? Most likely in the 5-10 % range. The range of outcomes should be presented.
We thank the reviewer for this comment and have altered the revised version accordingly. We now display the mean of nucleotide frequencies based on the Illumina sequencings of three replicate as bar graphs (mean ± standard deviation) in the main figures without further digits. Detailed comparison of the Illumina reads replicates can be found in the corresponding Supplementary Figures (S3, S7, S10). As digits, we provide the mean efficiency ± standard deviation for *de novo* PAM generation and a selected single downstream edit (e.g. splice site mutation) in the main figures. For the range of outcome alleles, we present the translation of anticipated alleles and their frequencies in the main figures.

3. I'm not a fan of new jargon, such as the phrase 'inceptionist' in the title. This did not enhance scientific communication in my personal view.
We are very much in favor of this word creation but are happy to accept any decision of the editor.

Reviewer 3 Advance Summary and Potential Significance to Field...

A major limitation to generating mutations that model human disease-associated SNPs in cells or model organisms using base editors designed around Cas9 is the requirement for a PAM sequence 13-17 nt downstream of the desired edit site. A number of modifications to the base editor enzyme have been described that alter or relax the strict PAM requirement of Cas9, however none of these are perfect and there is a constant need for alternative strategies for precise genome engineering. This manuscript describes a new strategy, "Inception", that uses base editors to first generate a new PAM site, and then to use that site to make the desired edit, in a one-pot (actually, one embryo) reaction. The net effect is increase the number of nucleotides accessible for editing by a factor of about 1.6: now edits can additionally be made in a window 27-36 upstream of a canonical PAM site.

© 2023. Published by The Company of Biologists under the terms of the Creative Commons Attribution License (https://creativecommons.org/licenses/by/4.0/).

Using genes with known phenotypes in Medaka fish as an experimental paradigm, and injecting sgRNAs and base editor mRNAs into embryos at the one-cell stage, the authors demonstrate successful examples of sequential editing to generate a novel NGG PAM followed by editing to generate desired mutations. While the authors don't attempt to generate germline alleles for any of these edits, at this point there is sufficient proof in the literature that mosaic mutations can be passed through the germline that there is little doubt that these will too.

Reviewer 3 Comments for the Author...

The work represents a significant advance and merits publication as a technical report in Development, however the text is very brief and both the text and the figures contain many inconsistencies and omissions that make it difficult to follow and understand.

1) The authors should state more clearly at the outset in the main text (not just the methods) what their percentages refer to. For example, line 145: does 79.7% refer to the percentage of sequenced PCR amplicons containing the 1011A>G mutation, or the percentage of larvae in which that mutation was detected?
We thank the reviewer for raising this point and have ensured a clear description of percentages used in the text. When mean nucleotide (nt) frequencies (± standard deviation) are provided, these are based on total Illumina reads (three replicates each). The abundance of allele or codon frequencies (± standard deviation) are based on a CRISPResso2 allele frequency table output file with a cut-off at 0.2% read abundance of the respective sequence in each individual Illumina sequencing replicate. This is now clearly described throughout the text.

If it is the former, then how many amplicons from how many larvae were sequenced? Were the larvae that were sequenced chosen randomly, or were they the most phenotypically affected?
We thank the reviewer for these questions, which made us aware that this information was buried too deep in the materials and methods section. We now have incorporated the relevant information (numbers of embryos per pool and Illumina reads) at all appropriate positions throughout the text. Pools were selected randomly for control injections and based on phenotypic embryos for the inception injections.

2) In line 146, does 26.1% refer to the percentage of 1011A>G containing amplicons that also have the Gln333* mutation, or the percentage of all amplicons containing the Gln333* mutation?
In the initial manuscript the 26.1% corresponded to all *oca2*-inception alleles with a PTC in position of *oca2-step2* (based on CRISPResso2, cut off >0.2% abundance, old Figure S1). We noticed that providing total nucleotide changes in combination with codon changes within the Illumina sequencing results was confusing.
We now address both individually, i.e. in the main figures, the displayed nucleotide changes at the Illumina sequencing results always refer to nucleotide changes across all Illumina reads. The resulting anticipated allele frequencies (translated) are based on the CRISPResso2 allele frequency analysis and are displayed (new Figure 2D).

3) Fig. 2 includes a statement of "Inception efficiency" that is not explained until the very end of the text (line 196).
In the revised manuscript, we no longer calculate the inception efficiency.

Since the ultimate goal of the approach is to generate a specific targeted mutation (e.g. Q333* for oca2; splice acceptor loss for rx3) without generating non-synonomous mutations at the new PAM site, the "inception efficiency" percentage stated in Fig. 2 should be the efficiency with which this precise goal was accomplished, i.e. the percentage of amplicons with this mutation and no other non-synonomous mutation. This does not appear currently to be the case, if, for example 26% of oca2 amplicons have a PTC, the inception efficiency can't be 75.5%.

The term inception efficiency seemed confusing and we have removed it throughout the revised version.
From all reviewer comments we realized that we were not sufficiently clear to delineate the fundamental aim. We now avoid the misleading impression that we aimed for exclusively introducing the Q333* codon mutation in *oca2* or the exclusive splice acceptor site removal in *rx3*. When disrupting a gene function by introduction of a PTC or splice acceptor manipulation, further

© 2023. Published by The Company of Biologists under the terms of the Creative Commons Attribution License (https://creativecommons.org/licenses/by/4.0/).

codon changes are at least negligible. We make that clear now in the revised version of the manuscript.

4) Since the new PAM site generated in oca2 is one nucleotide upstream of an existing PAM site (AGG), the authors have missed the opportunity to compare the efficiency of a conventional one-step C-to-T base editing event to the efficiency of Inception.
We thank the reviewer for this valuable point. Actually, we recently have published the efficiency of this very "conventional one-step C-to-T base editing event" (Cornean et al., 2022, eLife, doi: 10.7554/eLife.72124). Here this very guide RNA injected in combination with the ancBE4max leads to the introduction of the Q333* PTC in 65.3 % of cases (Figure 3a in Cornean et al., 2022). We now include this statement in the outline of the *oca2* experiment "Using base editors, we recently demonstrated that in oca2, non-synonymous changes of Threonine 332 (T332) as well as the introduction of a PTC (Glutamine>PTC, Q333*) resulted in substantial loss of pigmentation (Cornean et al., 2022)".

5) The authors comment (line 150-151) that the two base editors appear to show higher activity at their respective intended target sites. This is a completely mysterious finding that should be explained, however the only controls that are provided all involve injecting both base editors (A-to-G and C-to-T) with one or the other sgRNA. Controls should be performed in which both sgRNAs are injected with single base editors.
We agree with the reviewer in finding this behavior of the base editors noteworthy: Base editing efficiency is strongly context dependent, i.e. the nucleotide composition of the target site has a strong impact on individual nucleotide editing efficiencies. More precisely, the dinucleotide context, i.e. the influence of the preceding nucleotide on the editing efficiency can explain the observed differences in editing of both base editors at each target site. In the case of the ABE8e, a preceding adenine as present in the E331/T332 codons (i.e. *oca2* step 2) can drastically reduce the editing efficiency as we have shown earlier in Cornean et al., 2022 (eLife, doi: 10.7554/eLife.72124). The same is true for the canonical base editing window (*oca2* step 1) in which the cytosine of codon A337 (GCA) is preceded by a guanine which reduces the efficiency of the ancBE4max (Cornean et al., 2022). Thus, the base editors perform more efficiently on their respective intended target site. We have now included this explanation in the main text. The same AA dinucleotide influence on the ABE8e efficiency can be seen in the canonical base editing window of *kcnh6a* – here c.1517A>G is poorly edited (3.4%±1%), although contained within the base editing window.
Further, we have performed and included the suggested ABE- and CBE-control injections for *oca2* in new Figure 2 and the corresponding supplementary Figures S2.

6) The text and Table S2 refer to "inception sgRNAs" (in the case of oca2) and "inception-adjusted sgRNAs" (in the case of rx3 and kcnh6a). What is the difference between these? Why is the experimental condition for oca2 and rx3 "inception" and the experimental condition for kcnh6a "inception-adjusted"? Also, what is the difference between "newPAM control" (Table S1 and Table S2 for oca2) and "canonical PAM control" for Table S2 rx3 and kcnh6a? If these are real differences they need to be carefully explained, but if they are actually the same, they should be named the same way to spare your readers a great deal of confusion.
We realized that naming of the guide RNAs and use of the word "inception" for the two step process was misleading. To clarify we now use the following naming throughout the manuscript:

- guide RNAs that introduce the *de novo* PAM (step1) are called:
  - *oca2-step1, kcnh6a-step1, rx3-step1*
- guide RNAs that edit at the second site (step2) are called:
  - *oca2-step2, kcnh6a-step2-wt, kcnh6a-step2-adjusted, rx3-step2-adjusted*
- The term "inception" now exclusively refers to injections containing all: the base editor(s), step1 and step2 guide RNAs. All control experiments lacking any of the components are named accordingly: step1 control, step2 control, ABE control, CBE control

Alongside with the expansion of the main text, we stress the necessity to adjust the sequence of the *kcnh6a*- and *rx3-step2* guide RNAs and include an explanation in the main text.

© 2023. Published by The Company of Biologists under the terms of the Creative Commons Attribution License (https://creativecommons.org/licenses/by/4.0/).

7) In general, the section of the main text describing the kcnh6a approach is very confusing. What is the "inception-original cr/trRNA" mentioned at the bottom of Table S2 and in Fig. S3? Which data does it correspond to in Table S3? In the methods, the kcnh6a- inception-original crRNA and the kcnh6a-inception adjusted crRNA appear to have the identical sequence: TCTCATTGGATTACTGAAGg[CAG].
We thank the reviewer for critical reading. Indeed, there was a copy/paste error of the guide RNA sequences which is fixed now. We made sure that the renaming of the guide RNAs in the text, figure legends and supplementary items is unambiguous now as detailed above.

8) In table S2, information should be provided on the number of oca2-edited larvae that had with oca2 phenotypes (mosaic eyes).
In the revised manuscript, the numbers of pooled phenotypic embryos per replicate that were sent for Illumina sequencing are stated in the Material & methods section as well as in the main text now.

9) The term "unedited codons" which appears in Table S3 should be mentioned in the main text and it should be explained why edited and unedited codon percentages do not add up to 100%
In the revised manuscript, we no longer analyze codons individually but refer to codon changes in the context of the respective altered alleles. Thus, we have removed the old table S3 and provide the resulting translations in the allele frequency table (old Figure S1, now split per locus in Fig. S2, S6, S9). We as well no longer use the term "unedited" but compare the edited alleles with the frequency of wild-type reads, if found in the Illumina sequencing data.
The codon analysis is based on the allele frequency table generated by the CRISPResso2 tool and contains all alleles with a frequency >0.2% abundance. Thus, the sum of all these abundances do not add up to 100%.

10) Table S3 section on rx3 editing is very confusing. It contains no information on the efficiency of de-novo PAM generation frequency, although it is stated as 42% in the text and Fig. 2, and it contains no information on the mutation frequency at the target splice acceptor site, stated as 38% in the text and Fig. 2. The methods states that genomic DNA, not cDNA was extracted from injected embryos, so it's not clear why intron sequence was not captured. Intron sequence including both edit sites should be included for all three conditions (inception, newPAM control and rx3 inception control).
It is correctly stated that genomic DNA was extracted from all injected embryos and therefore intron sequences were captured for the *rx3* locus in the original manuscript. However, the old Table S3 provided translation only of coding sequences and left the intronic region "blank". As mentioned above we have removed Table S3 to enhance clarity and have now updated the allele frequency table of *rx3* with the resulting translation in the exonic part of the *rx3* amplicons (Figure S10).

Minor comments:

1) This reviewer does not like the term "editants" which, unlike "morphant" and "CRISPant" is too vague to be a useful short-form.
We used this term since it was already coined in previous publications (Thumberger et al. 2022, Cornean et al., 2022).

2) The term "literally" (line 187) should be removed. Scientific reporting should always be literal. Paradoxically, in this case "literally devoid" is meant to mean "almost but not quite entirely devoid…" (2/57 embryos had heart phenotypes).
This statement referred to the Illumina sequencing results of the *kcnh6a-step2-adjusted* control in which no editing could be found. This is still the case when analyzing all three replicates. We now changed the wording to omit "literally": "The kcnh6a-step2-adjusted did not result in scorable editing events (0.1 % ± 0.1 %) in the absence of the canonical editing event (Fig. S6; 3 pools of 5, 91445 reads total)".

3) The title of the paper ("The inceptionist's guide...") makes it sound like a review rather than an original work. Recommend changing to "de-novo PAM generation to reach initially inaccessible target sites using base editing".

© 2023. Published by The Company of Biologists under the terms of the Creative Commons Attribution License (https://creativecommons.org/licenses/by/4.0/).

We thank the reviewer for sharing their concern. We followed the suggestion and have inverted the title accordingly.

Second decision letter

MS ID#: DEVELOP/2022/201115

MS TITLE: De novo PAM generation to reach initially inaccessible target sites - the inceptionist's guide to base editing

AUTHORS: Kaisa Pakari, Joachim Wittbrodt, and Thomas Thumberger

You will be pleased to hear that the referees are now happy with your revisions. The referees' comments are appended below, or you can access them online: please go to BenchPress and click on the 'Manuscripts with Decisions' queue in the Author Area.

Before we proceed to publication, I want to pass on a plea from one ofthe reviewers about the introduction of new jargon - specifically "inception". The reviewer feels vey strongly the the field is being overwhelmed with new terminology at the detriment to clarity and understanding. The referee considers your study doesn't need any new terms and that the use of "inception" really does not help readers understand the approach. I do understand this perspective, however I'm not going to make any changes a condition for publication - its in your hands to decide what you prefer to do.

Reviewer 1

Advance summary and potential significance to field

The manuscript by Pakari, Wittbrodt and Thumberger presents a very exciting idea of extending the repertoire of base editable sites by using adenine base editors to introduce ("incept") new PAM sites. This work is highly significant and has tremendous potential.

Comments for the author

Thank you for thoroughly addressing all comments.

Reviewer 2

Advance summary and potential significance to field

CRISPR systems have restricted sequence access due to their traditional PAM sequences. This paper describes a clever, dual CRISPR base editing strategy to add a new PAM nearby an existing site to make additional targeted gene alleles. They use the medaka, but this approach is likely well-suited for any animal model used in development.

Comments for the author

The authors have fully addressed my concerns. This is a nice manuscript and a method that will have impact in many model systems.

© 2023. Published by The Company of Biologists under the terms of the Creative Commons Attribution License (https://creativecommons.org/licenses/by/4.0/).

Reviewer 3

Advance summary and potential significance to field

A major limitation to generating mutations that model human disease-associated SNPs in cells or model organisms using base editors designed around Cas9 is the requirement for a PAM sequence 13-17 nt downstream of the desired edit site. This manuscript describes a new strategy, termed “Inception”, that expands the targetable genome using base editors to sequentially generate a new PAM site, and then to use that site to make the desired edit. Using genes with known phenotypes in Medaka fish as an experimental paradigm, and injecting sgRNAs and base editor mRNAs into embryos at the one-cell stage, the authors demonstrate successful examples of sequential editing.

Comments for the author

The authors have addressed my criticisms in their revised manuscript. The current manuscript is easier to read and understand, and includes the appropriate controls for all three targeted genes. In general there is more consistency in the naming of the various components (base editors and guides), making the whole process easier to follow. I feel that the revised manuscript is now appropriate for publication as a technical report in Development.

Second revision

Author response to reviewers' comments

We thank the reviewers for their critical reading and support for improving our manuscript.

Third decision letter

MS ID#: DEVELOP/2022/201115

MS TITLE: De novo PAM generation to reach initially inaccessible target sites - the inceptionist's guide to base editing

AUTHORS: Kaisa Pakari, Joachim Wittbrodt, and Thomas Thumberger
ARTICLE TYPE: Research Report

I am happy to tell you that your manuscript has been accepted for publication in Development, pending our standard ethics checks.

© 2023. Published by The Company of Biologists under the terms of the Creative Commons Attribution License (https://creativecommons.org/licenses/by/4.0/).
